# Revision of the Palaearctic species of the *Merodon desuturinus* group (Diptera, Syrphidae)

**DOI:** 10.3897/zookeys.771.20481

**Published:** 2018-07-05

**Authors:** Ante Vujić, Snežana Radenković, Laura Likov

**Affiliations:** 1 Department of Biology and Ecology, University of Novi Sad, Trg Dositeja Obradovića 2, 21000 Novi Sad, Serbia

**Keywords:** *Merodon auripilus*, *Merodon cuthbertsoni*, *Merodon murorum*, *Merodon neolydicus* nom. n.

## Abstract

This revision of material belonging to the *Merodon desuturinus* group from the Palaearctic Region resulted in the delimitation of four species: *Merodon cabanerensis* Marcos-García, Vujić & Mengual, 2007; *Merodon desuturinus* Vujić, Šimić & Radenković, 1995; *Merodon neolydicus* Vujić, **nom. n.**; and *Merodon murorum* Fabricius, 1794. *Merodon murorum* is redescribed. A neotype for *Merodon auripilus* Meigen, 1830 is designated, which is a new junior synonym of *Merodon murorum*. The related Afrotropical species *Merodon cuthbertsoni* Curran, 1939 is re-evaluated and compared to its sibling Palaearctic taxon *Merodon desuturinus*. An identification key for the *Merodon desuturinus* group is provided.

## Introduction

The genus *Merodon* Meigen, 1803 (Diptera, Syrphidae) comprises more than 160 species distributed across the Palaearctic and Afrotropical Regions (Ståhls et al. 2009). The Mediterranean Basin hosts the highest diversity with more than 110 species, most probably due to high variety of bulb plants that are larval hosts of this phytophagous genus (Ricarte et al. 2008, Andrić et al. 2014).

Hurkmans (1993) conducted the first published revision of part of this genus, analysing 61 species (only those with tapering abdomens) classified into eleven groups. In the last decade, a number of papers have been published on particular species groups, such as *aureus*, *melanocerus*, *nanus*, *natans*, *nigritarsis*, *ruficornis* (Milankov et al. 2008a, Francuski et al. 2009, 2011, Vujić et al. 2012, 2013, 2015, Šašić et al. 2016, Radenković et al., 2018). The Mediterranean fauna has been frequently studied (Mengual et al. 2006, Marcos-García et al. 2007, 2011, Petanidou et al. 2011, Radenković et al. 2011, Ricarte et al. 2012, Ståhls et al. 2009, 2016, Vujić et al. 2007, 2011), whereas the Afrotropical Region with less than ten recognised species (Pape and Thompson 2013), has received less attention (Radenković et al. 2018).

Initial research on the phylogeny of the *Merodon* genus was conducted by Mengual et al. (2006). Based on analysis of COI sequences of the Iberian species, they defined four well-supported groups: *desuturinus*, *albifrons*, *nigritarsis*, and *aureus*.

One in particular, the so-called “*desuturinus* group” of range-restricted species, is of special conservation interest as it has members in both the Palaearctic and Afrotropical Regions. Vujić et al. (1995) described an endemic species, *Merodon desuturinus*, from the high mountains of the Balkan Peninsula. Later, Marcos-García et al. (2007) discovered a related species, *M. cabanerensis*, from central Spain. Another species was recognized by Hurkmans (unpublished manuscript, cited in Milankov et al., 2008b) as *M. lydicus* Hurkmans, which was recorded in the Eastern Mediterranean. This latter species is formally described here as *M. neolydicus* Vujić, nom. n. One additional taxon belonging to the *desuturinus* group, *M. murorum* Fabricius, 1794, was uncovered and we redescribed it here.

Milankov et al. (2008b) detected low genetic variability in a population of *M. desuturinus* and demonstrated that this taxon represents an evolutionarily independent lineage among *Merodon* taxa.

Radenković et al. (2018) found new members of the *desuturinus* group in South Africa, which are related to *Merodon melanocerus* Bezzi, 1915. Those records represent the first detailed characterisation of *Merodon* species in the Afrotropical Region using morphological and molecular data.

The aim of this paper is to present a revision of the Palaearctic species of the *Merodon desuturinus* group in order to clarify its taxonomy with the support of morphological characters, and to present an identification key for the adults of the species within this group.

## Materials and methods

This study is based on the examination of all available material of the *Merodon desuturinus* species group (published and unpublished data), which has been deposited in the museums, universities and private collections listed below. The following acronyms for museums and entomological collections are used in the text:

**AEU** University of the Aegean, Mytilene, Greece

**AMNH** American Museum of Natural History, New York

**BMNH** Natural History Museum, London

**CEUA** Colección Entomológica Universidad de Alicante, Spain

**FSUNS** Faculty of Science, University of Novi Sad

**KBIN** Royal Belgian Institute for the Natural Sciences, Brussels, Belgium

**MNHN** Musee National d’Histoire Naturelle, Paris, France

**MNMS** Museo Nacional de Ciencias Naturales, Madrid, Spain

**MZH** Finnish Museum of Natural History, University of Helsinki, Finland

**NHMB** Prirodnjački Muzej Beograd, Serbia

**NHMW** Naturhistorisches Museum Wien, Austria

**NMNL** National Museum of Natural History Naturalis, Leiden, Netherlands

**TAU** Tel Aviv University, Israel

**ZMHB** Zoologisches Museum of the Humboldt University, Berlin, Deutschland

**ZMUC** Zoological Museum, University of Copenhagen, Denmark

**WML** World Museum Liverpool, UK

**coll. C. Palmer** Private collection of Chris Palmer, United Kingdom

**coll. E. Gilasian** Private collection of Ebrahim Gilasian, Iran

**coll. M. Taylor** Private collection of Mike Taylor, United Kingdom

**coll. V. Weyer** Private collection of Guy Van de Weyer, Belgium

The characters used in the key, descriptions, and drawings follow the terminology established by Thompson (1999), except for the term “pleuron”, which follows McAlpine (1981), and those relating to male genitalia are according to Marcos-García et al. (2007). Colour characters are described from dry-mounted specimens. Male genitalia were stored in microvials containing glycerol after clearing in warm 10% potassium hydroxide (KOH) for a few minutes and neutralising in acetic acid for 5–10 seconds.

The following abbreviations are used: **f** = female, **m** = male.

All information on the specimens (locality, collector, coordinates, etc.) is presented under the description of the respective examined material. The capture locations (geographical coordinates) were entered into the GenGIS (v2.5.1) software to generate the distribution map (Parks et al. 2013).

Diagnoses of species were made according to unique characters attributable to the group, complex, and species considered here, and also to combinations of characters that enabled taxa to be distinguished and recognised. The type material of the included species was examined by Ante Vujić. Drawings were made with an FSA 25 PE drawing tube and digital photographs were taken with a Leica DFC 320 digital camera, both of which were attached to a Leica MZ16 binocular microscope.

## Results

### Diagnostic characters and diversity of the *Merodon desuturinus* species group

**Figure 1.**
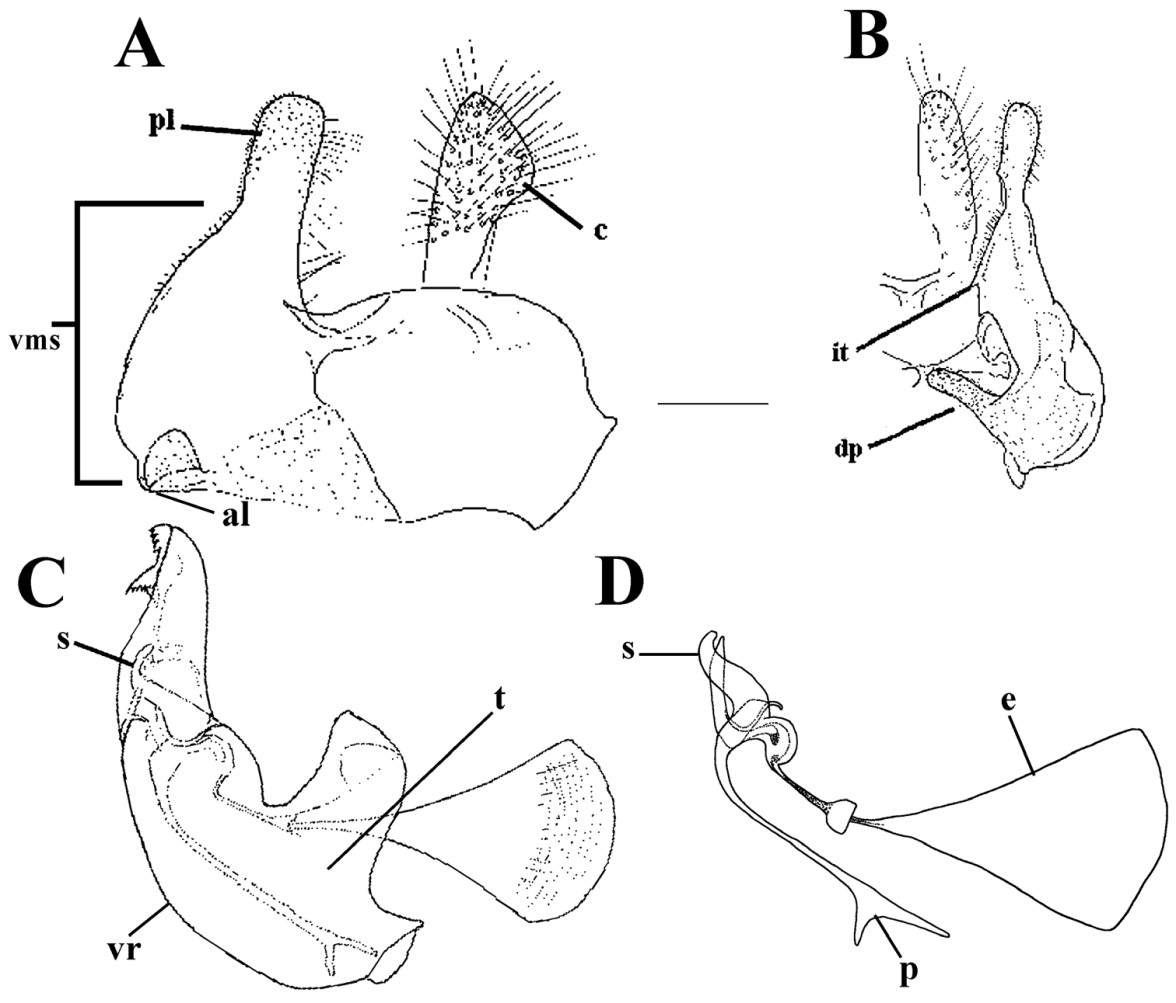
*Merodon neolydicus* Vujić, nom. n. male genitalia. **A** Epandrium, lateral view **B** Epandrium, ventral view **C** Hypandrium, lateral view **D** Aedeagous. Abbreviations: **al**-anterior surstyle lobe, **pl**-posterior surstyle lobe, **c**-cercus, **e**-ejaculatory apodeme, **p**-phallapodeme, **s**-lateral sclerite of aedeagus, **it**-inner thorn on medial part of surstylus, **dp**-distal prolongation on anterior surstyle lobe, **vms**-ventral margin of surstylus, **vr**-ventral ridge of theca, **t**-theca. Scale bar: 0.2 mm.

The *M. desuturinus* species group sensu Mengual et al. (2006) is characterised by the following adult morphological characters: posterior side of mesocoxa with pile; anterior surstyle lobe with a curved distal prolongation (dp in Figs 1B, 9B, 11B, 12B, 14B); the specific shape of the lateral sclerite of the aedeagus (gradually tapered, with the tip curved downwards) is the main synapomorphic character that connects all species from the group (s in Figs 1C, D, 9C, 11C, 12D, 14C, 16) (Vujić et al. 1995, Milankov et al. 2008b). The *M. desuturinus* species group is closely related to the *albifrons* group (Mengual et al. 2006), which has been designated as an *albifrons*+*desuturinus* clade in Radenković et al. (2018).

The *M. desuturinus* group consists of two clearly separate lineages, Palaearctic and Afrotropical based on both adult morphological and molecular data (Radenković et al. 2018). The main morphological diagnostic character that distinguishes these two lineages is the presence of a strong dense yellow to red brush of pile on the metatrochanter of Afrotropical species, which Palaearctic taxa lack. Besides the taxon *M. desuturinus*, the Palaearctic lineage of this species group includes three additional species, one western Mediterranean endemic (*M. cabanerensis*) and two species presented here.

**Figure 2.**
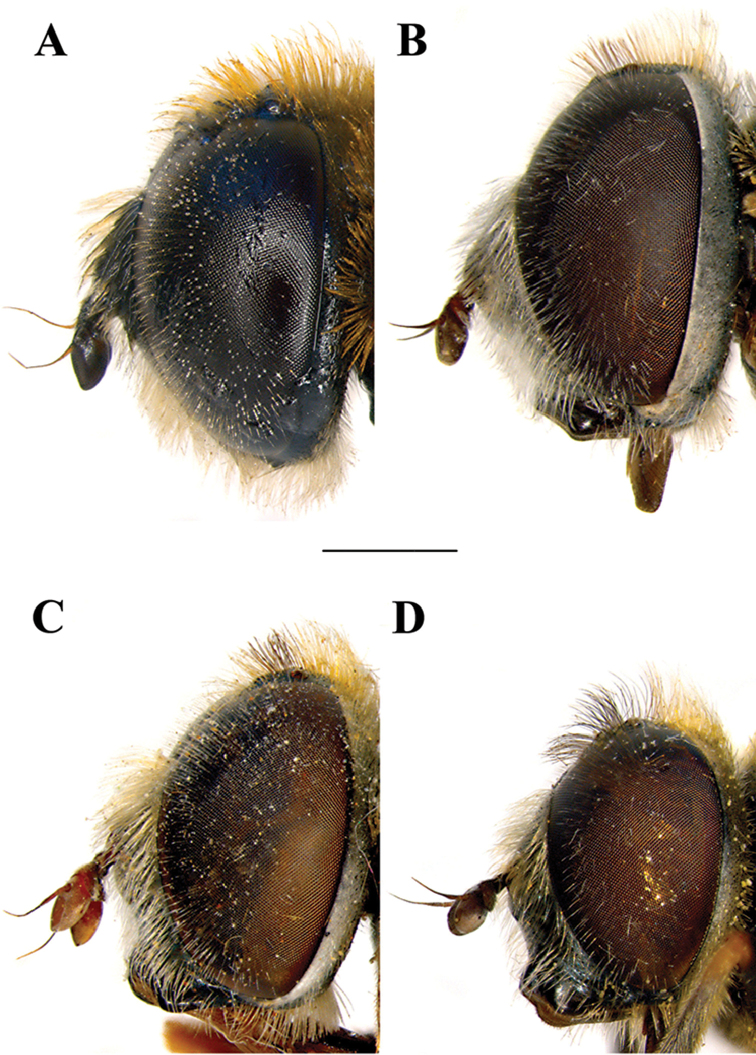
Male head, lateral view. **A** *Merodon planifacies* **B** *Merodon neolydicus* Vujić, nom. n. **C** *Merodon murorum* **D** *Merodon desuturinus*. Scale bar: 2 mm.

The Afrotropical lineage of the *M. desuturinus* group (Radenković et al. 2018) comprises nine taxa arising from a revision of the *M. melanocerus* subgroup (*Merodon capensis* Hurkmans, 2018; *Merodon commutabilis* Radenković & Vujić, 2018; *Merodon drakonis* Vujić & Radenković, 2018; *Merodon flavocerus* Hurkmans, 2018; and *Merodon melanocerus* Bezzi, 1915) and the *Merodon planifacies* subgroup (*M. planifacies* Bezzi, 1915, *Merodon stevensoni* Curran, 1939 and one undescribed species so far) with a reduced oral margin covered by microtrichia as a clear apomorphic character (Fig. 2A). The other Afrotropical species of the group, *Merodon cuthbertsoni* Curran, 1939, which is morphologically related to *M. desuturinus*, is redefined here.

### General description of the *Merodon desuturinus* species group

**Figure 3.**
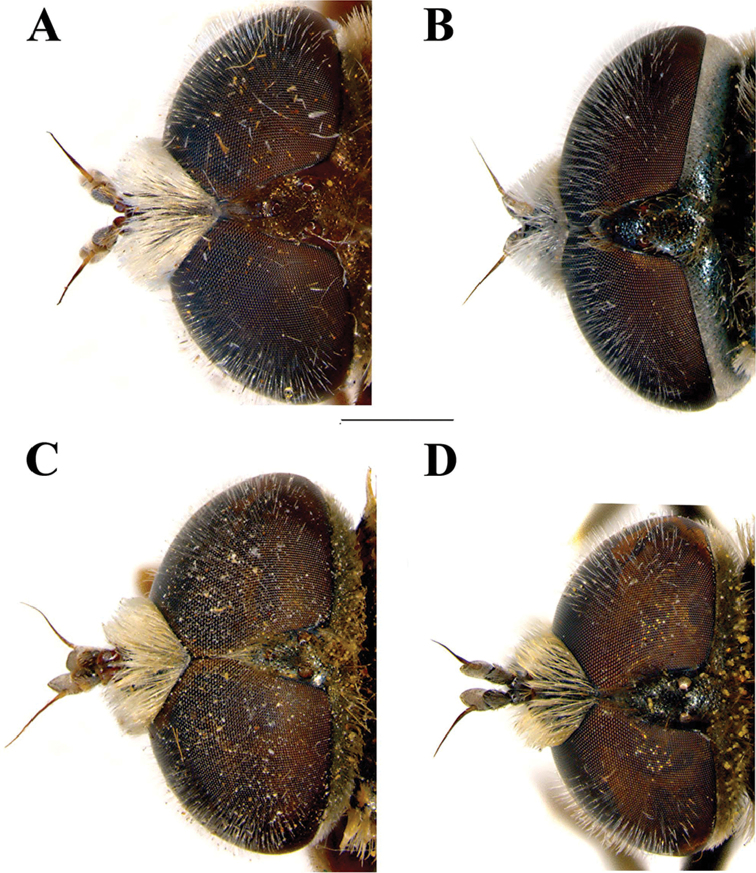
Male head, dorsal view. **A** *Merodon flavocerus* **B** *Merodon neolydicus* Vujić, nom. n. **C** *Merodon murorum* **D** *Merodon desuturinus*. Scale bar: 2 mm.

**Figure 4.**
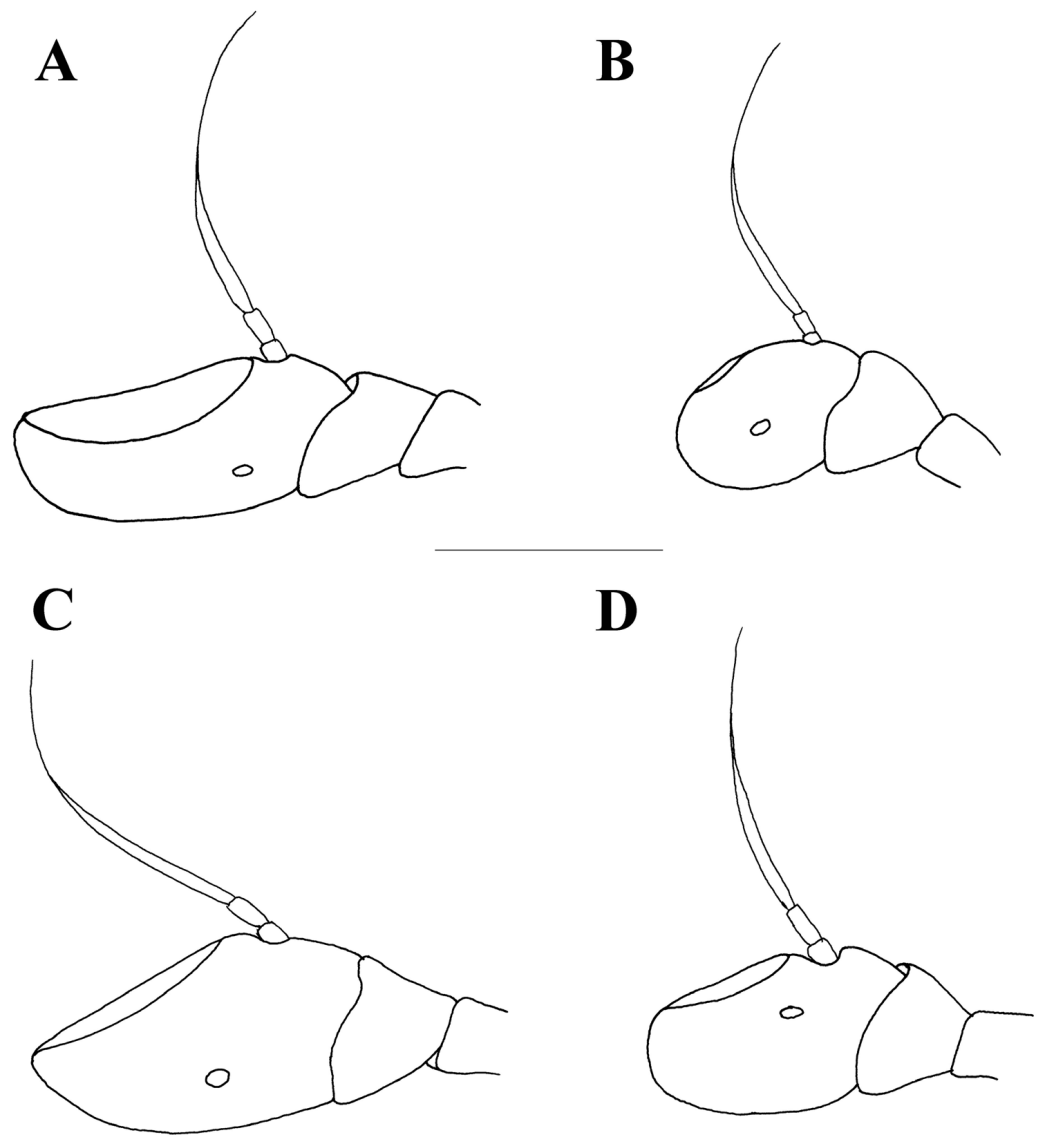
Male antennae, lateral view. **A** *Merodon flavocerus* **B** *Merodon desuturinus* **C** *Merodon murorum* **D** *Merodon neolydicus* Vujić, nom. n. Scale bar: 1 mm.

Male. *Head* (Figs 2, 3, 4): Antenna (Fig. 4) usually dark brown; basoflagellomere generally short, as long as broad (except in *M. flavocerus* where it is light brown and longer), concave dorsally, with acute apex; arista light brown to dark brown, thickened basally, 1.5-2 times longer than basoflagellomere, covered with dense brown microtrichia. Face covered with long whitish yellow pile, except on median bare vitta that occupies 1/4 width of face. Frons black, often with bronze film and indistinct microtrichia that, at face level, follow a narrow line along the eye margin. Vertical triangle isosceles (Fig. 3), black (brown-red in *M. flavocerus*) and shiny (except at anterior end covered with microtrichia), predominantly covered with long, black, thick pile, except at posterior end with light yellow pile. Eye pile dense, as long as scape, often pale, but can be darker dorsally. Occiput covered with whitish yellow pile, dorsally with metallic, bluish or bronze lustre; white microtrichia from upper eye corner as a narrow line dorsally, becoming dense and wide laterally and ventrally, occupying the lower 2/3 of occiput.

**Figure 5.**
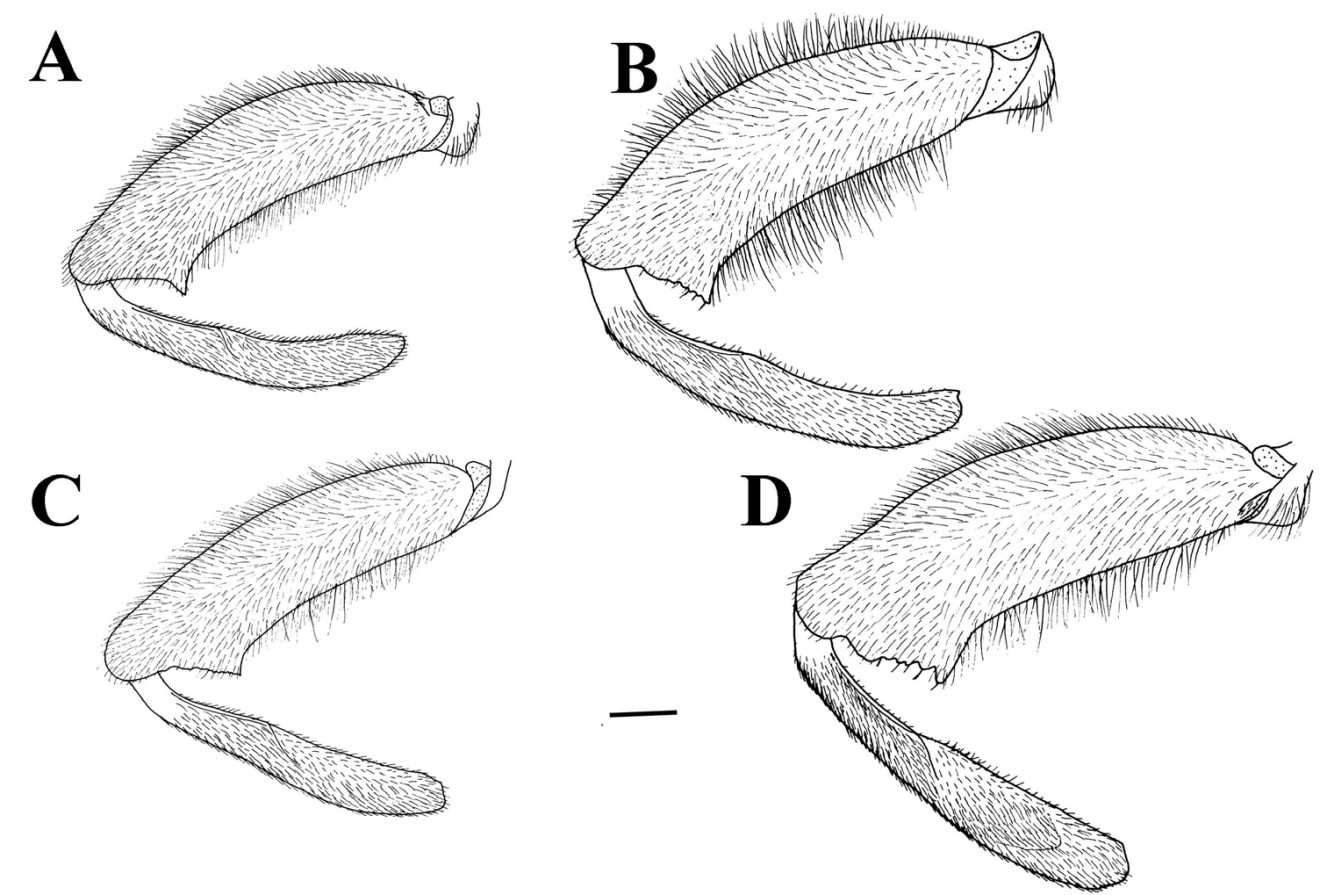
Metaleg, lateral view. **A** *Merodon neolydicus* Vujić, nom. n., male **B** *Merodon murorum*, male **C** *Merodon neolydicus* Vujić, nom. n., female **D** *Merodon murorum*, female. Scale bar: 1 mm.

*Thorax* (Fig. 5): Scutum and scutellum black with bronze lustre (in *M. flavocerus* postpronotum and posterior rim of scutellum pale yellow), covered with relatively long (as long as or a little longer than basoflagellomere), dense, erect, more or less branched and usually yellow pile (in *M. capensis* and *M. commutabilis* mixed with black pile); presence of microtrichia variable (from well-developed in *M. drakonis* to absent in *M. capensis*). Pleuron often covered with grey-green microtrichia (lacking in *M. flavocerus*) and the following parts with long yellow pile: posterior part of anterior anepisternum, posterior anepisternum (except anteroventral part), anepimeron, metasternum, and anterior, posterodorsal and posteroventral parts of katepisternum; katatergum with dense, erect, short, yellowish or light brown pile. Wing hyaline, with dense microtrichia and light brown to dark brown veins. Calypter yellow. Haltere with brown pedicel and yellow to brown capitulum. Legs usually dark brown-black (light brown in *M. flavocerus* and *M. murorum*), except in some cases for paler knees and paler bases and apexes of tibiae; colour of tarsi varies. Metatrochanter lacks processes, covered with yellow to orange pile. Metafemur moderately thickened and straight or slightly curved (Fig. 5). Metatibia with inconspicuous apical, anteroventral spur and indications of a posteroventral spur. Pile on legs predominantly yellow, except for some short black pile on tarsi dorsally.

**Figure 6.**
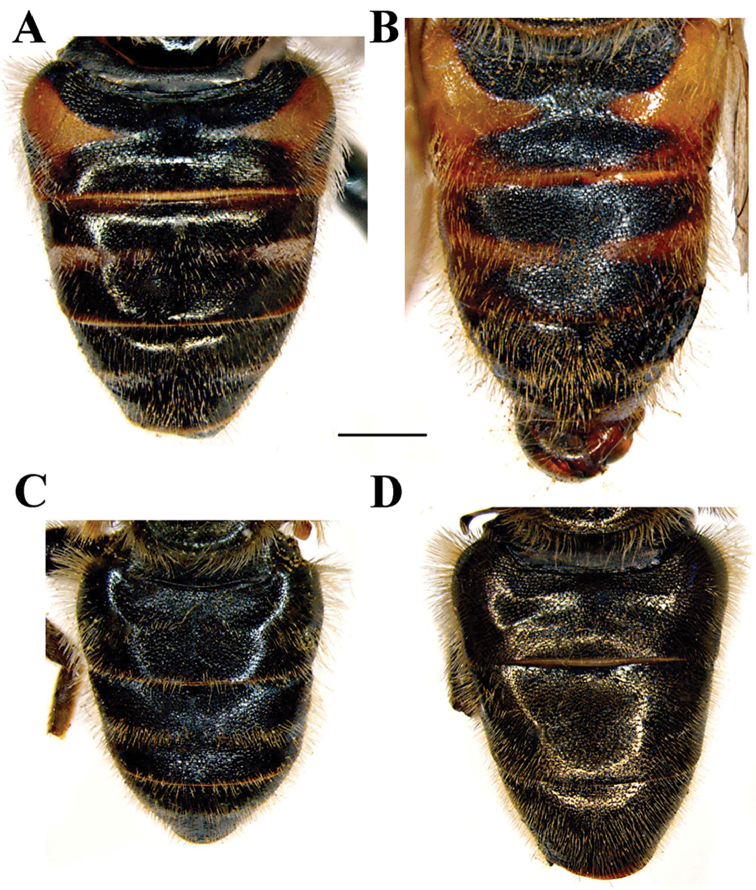
Male abdomen, dorsal view. **A** *Merodon neolydicus* Vujić, nom. n. **B** *Merodon murorum* **C** *Merodon desuturinus* **D** *Merodon cabanerensis*. Scale bar: 2 mm.

*Abdomen* (Fig. 6): Black with bronze reflections, slightly tapering, as long as mesonotum. Terga 2-4 black with more or less distinct transverse fasciae of white microtrichia interrupted in the middle (can be connected on tergum 4); tergum 2 in some taxa with pair of antero-lateral orange maculae (lacking in *M. capensis*, *M. cuthbertsoni*, *M. commutabilis*, *M. cabanerensis*, and *M. desuturinus*, all of which have dark terga), or areas covered with long, dense, erect, yellow pile; pilosity on lateral sides of terga long, erect and whitish, adpressed on central parts, and white on mictrotichose transversal fasciae, posterior 2/3 of tergum 4 and also on the posterior margin of terga 2-3 of most of the species (otherwise black). Sternum dark brown (except in *M. flavocerus* in which it is yellow) and shiny, covered with very long, pale yellow pile.

*Male genitalia*: Posterior surstyle lobe triangular, usually pointed apically (pl in Figs 11A, 12A, 14A, 15); ventral margin of surstylus straight (vms in Fig. 1A) or convex (vms in Fig. 14A); anterior surstyle lobe bent inwards; median part of surstylus with one or two inner thorns (it in Figs 1B, 9B, 11B, 12B, 14B, 15D); cercus elongated (c in Figs 1A, 9A, 11A, 12A, 14A). Hypandrium with broad theca (as in Figs 9C, 12D: t). Lateral sclerite of aedeagus narrow, gradually tapered, with the tip curved downwards (s in Figs 1C, 9C, 12D, 14C, 16A).

**Figure 7.**
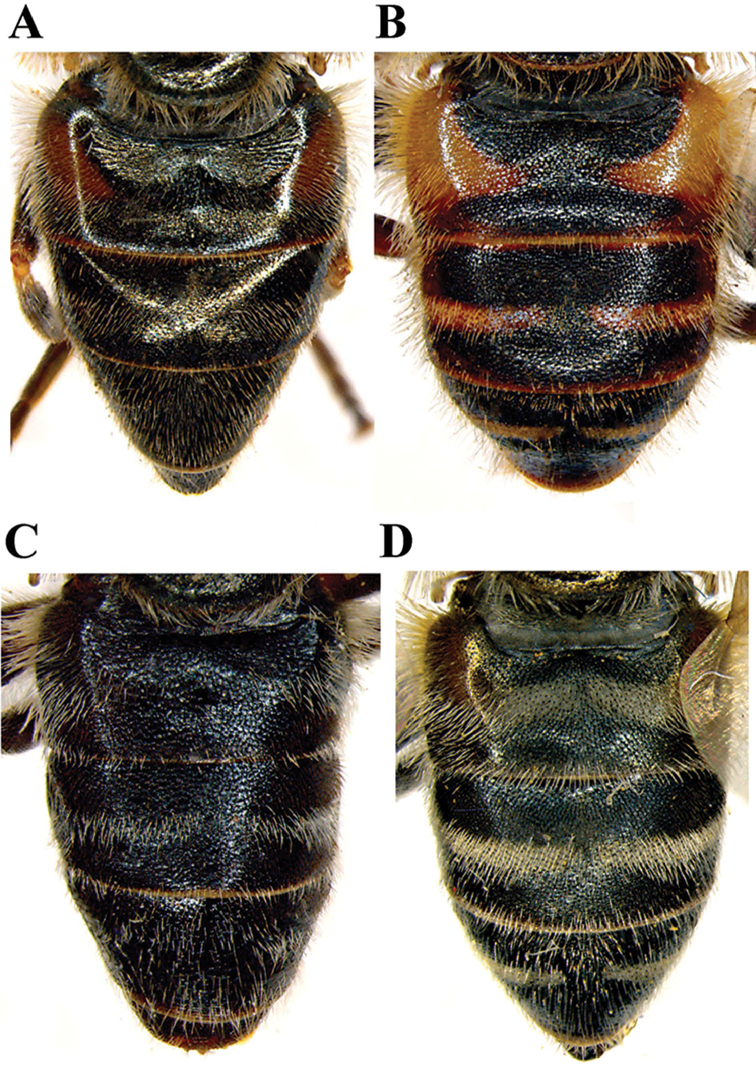
Female abdomen, dorsal view. **A** *Merodon neolydicus* Vujić, nom. n. **B** *Merodon murorum* **C** *Merodon desuturinus* **D** *Merodon commutabilis*. Scale bar: 2 mm.

**Figure 8.**
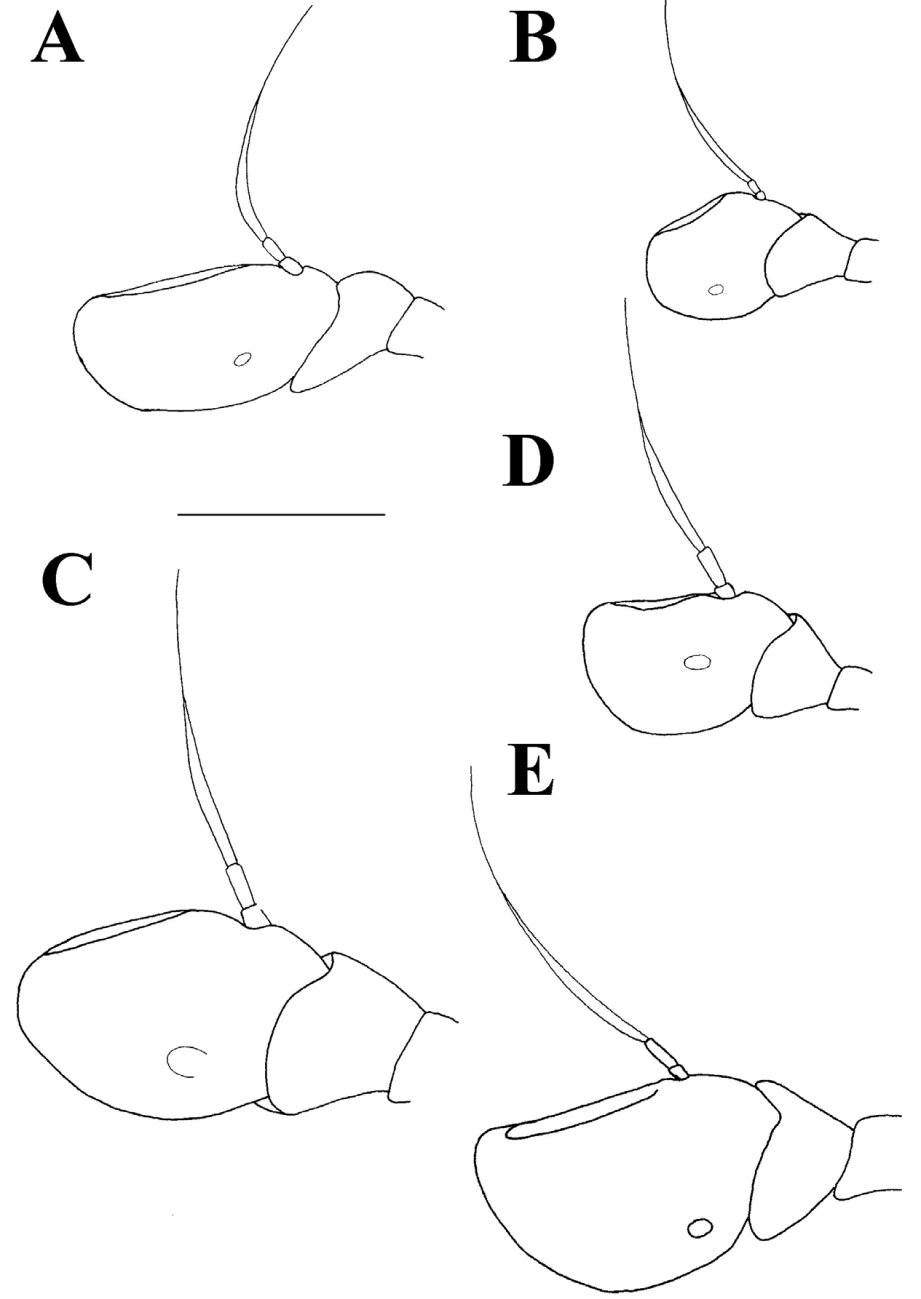
Female antennae, lateral view. **A** *Merodon flavocerus* **B** *Merodon desuturinus* **C** *Merodon murorum* **D** *Merodon neolydicus* Vujić, nom. n. **E** *Merodon commutabilis*. Scale bar: 1 mm.

*Female*: Similar to the male except for typical sexual dimorphism (Figs 5C, D, 7, 8).

*Length*: medium-sized species, body 10–13 mm, wing 6–9 mm.

### Species of the Palaearctic lineage of the *Merodon desuturinus* species group

#### Merodon cabanerensis

Taxon classificationAnimaliaDipteraSyrphidae

Marcos-García, Vujić & Mengual, 2007

Figs 6D , 9 , 10

##### Diagnosis.

**Figure 9.**
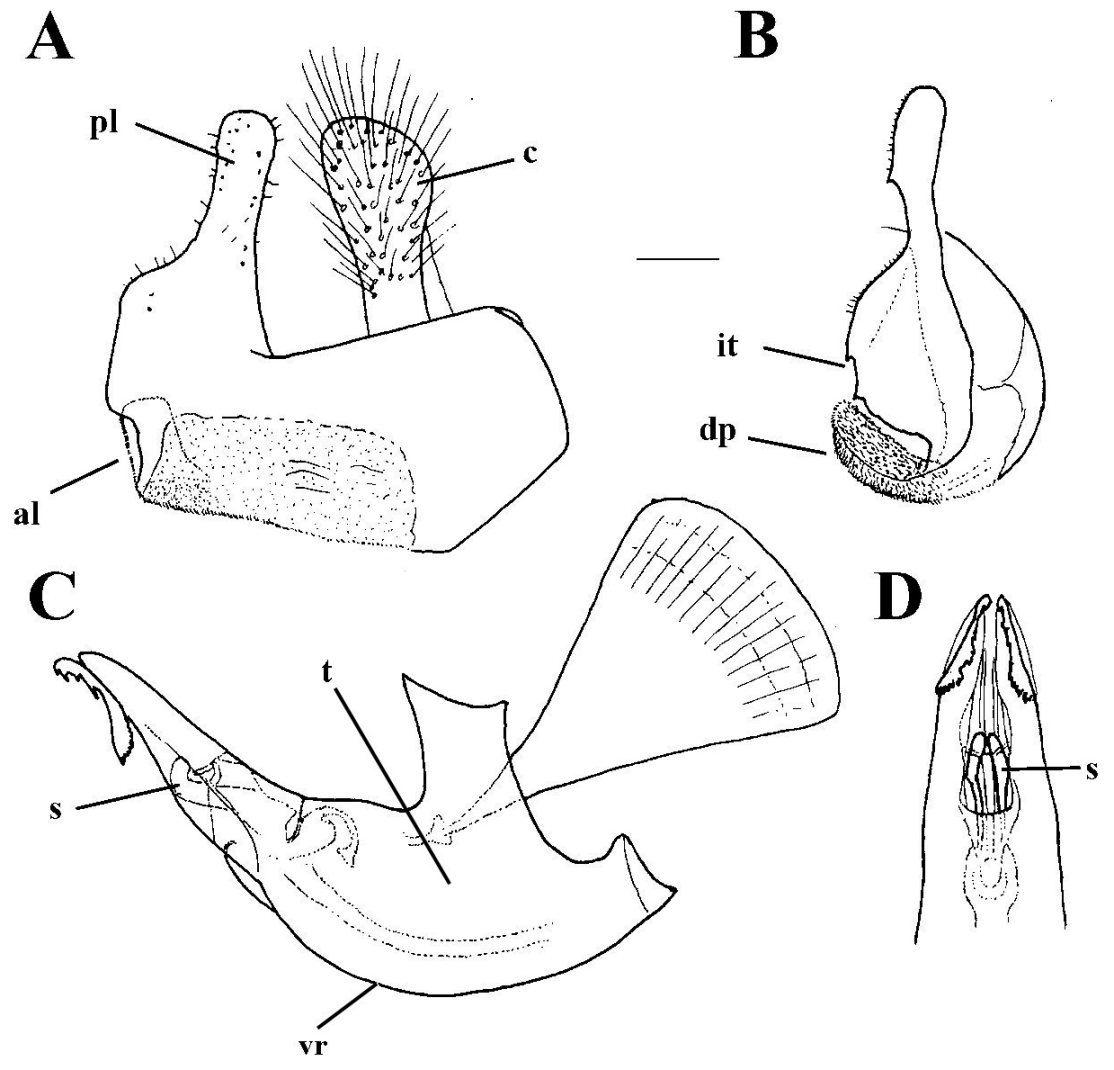
*Merodon cabanerensis* male genitalia. **A** Epandrium, lateral view **B** Epandrium, ventral view **C** Hypandrium, lateral view **D** Part of hypandrium, ventral view. Abbreviations: **al**-anterior surstyle lobe, **pl**-posterior surstyle lobe, **c**-cercus, **s**-lateral sclerite of aedeagus, **it**-inner thorn on medial part of surstylus, **dp**-distal prolongation on anterior surstyle lobe, **t**-theca, **vr**-ventral ridge of theca. Scale bar: 0.2 mm.

Small (8–11 mm) dark species with narrow abdomen, oral margin notched, evident, basoflagellomere small, 1-1.1 times as long as broad, legs dark. Terga mostly black, terga 2–4 with or without only a trace of transverse pair of microtrichose fasciae. Males: eye contiguity 8–10 facets long. Male genitalia with smooth thecal ridge, posterior surstyle lobe very narrow in apical half with parallel margins (Fig. 9). Similar to *M. neolydicus* nom. n., from which it differs by its smaller size (10–13 mm in *M. neolydicus* nom. n.), the shape of the male genitalia (Figs 1, 9), and its distribution (*M. cabanerensis* is found in the Western Mediterranean and *M. neolydicus* nom. n. occurs in the Eastern Mediterranean).

##### Examined material.

**Type material.** Holotype: male, pinned, in CEUA. Original label: “Spain, Ciudad Real, Canalejas, P. N. de Cabañeros, 39°24'17.03"N, 4°30'35.73"W, 19.iii.2004, leg. A. Ricarte”.

Paratypes: **Spain**: 1m+1f, Ciudad Real, Canalejas, P. N. de Cabañeros, 39°24'17.03"N, 4°30'35.73"W, 19.iii.2004, leg. A. Ricarte (CEUA); 1m, Ciudad Real, Canalejas, P. N. de Cabañeros, 39°24'17.03"N, 4°30'35.73"W, 19.iii.2004, leg. A. Ricarte (MNMS); 1m, Ciudad Real, Canalejas, P. N. de Cabañeros, 39°24'17.03"N, 4°30'35.73"W, 19.iii.2004, leg. A. Ricarte (FSUNS).

##### Additional material.

**Morocco**: 1m, Azilal, Ait Mhamed, 31°52'19.39"N, 6°29'6.72"W, 1700m, 26.iii.2013, leg. J. Dils, J. Faes (V.Weyer coll.).

##### Distribution.

**Figure 10.**
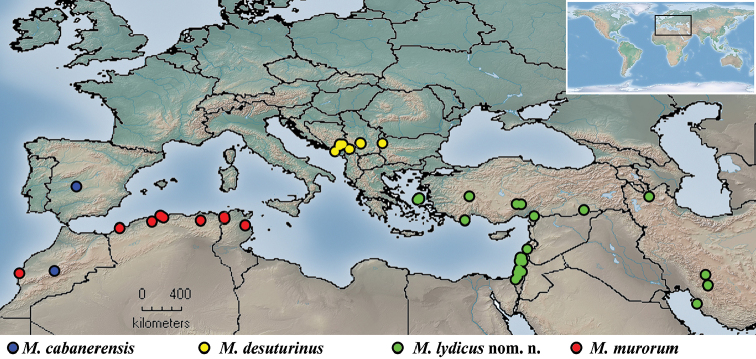
Distribution map of *Merodon cabanerensis*, *Merodon desuturinus*, *Merodon neolydicus* Vujić, nom. n., and *Merodon murorum*.

Iberian Peninsula and Morocco (Fig. 10).

#### Merodon desuturinus

Taxon classificationAnimaliaDipteraSyrphidae

Vujić, Šimić & Radenković, 1995

Figs 2D , 3D , 4B , 6C , 7C , 8B , 10 , 11 , 13B

##### Diagnosis.

**Figure 11.**
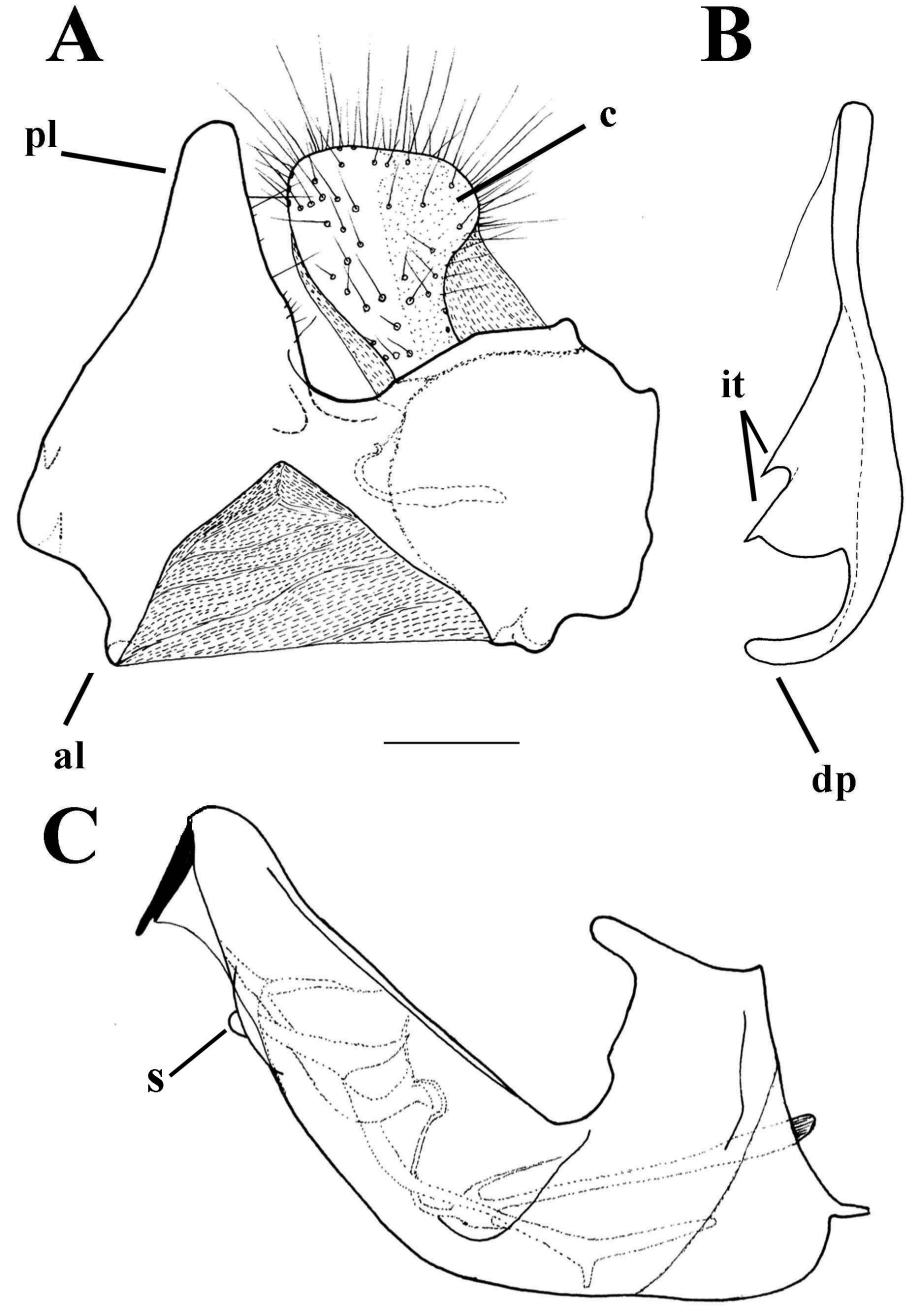
*Merodon desuturinus* male genitalia. **A** Epandrium, lateral view **B** Epandrium, ventral view **C** Hypandrium, lateral view. Abbreviations: **al**-anterior surstyle lobe, **pl**-posterior surstyle lobe, **c**-cercus, **it**-inner thorn on medial part of surstylus, **dp**-distal prolongation on anterior surstyle lobe, **s**-lateral sclerite of aedeagus. Scale bar: 0.2 mm.

Small (8–11 mm) dark species with dark legs; small and short basoflagellomere, 1–1.1 times as long as broad (Fig. 4B); oral margin notched, evident; narrow abdomen. Terga 2–4 with or without pair of narrow transversal microtrichose fasciae (Fig. 6C). Males: eyes almost touching (approaching) (Figs 3D, 13B). Male genitalia: hypandrium with smooth thecal ridge (Fig. 11C), posterior surstyle lobe elongated and triangular (Fig. 11A). Unique Palaearctic species with eyes almost touching (Fig. 3D). Female similar to *M. cabanerensis*, from which it can be separated by its distribution (*M. desuturinus* occurs in the Balkan Peninsula, whereas *M. cabanerensis* is distributed in the Iberian Peninsula).

##### Examined material.

**Type material**. Holotype: male, pinned, in NHMB. Original label: “Srbija, Kopaonik, Čukara-Jablanova ravan, 43°12'15.00"N, 20°50'13"E, 22.v.1993, leg. Vujić”.

Paratypes: **Serbia**: 18m+3f, Kopaonik, Čukara-Jablanova ravan, 43°12'15.00"N, 20°50'13"E, 1300-1400m, 22.v.1993, leg. Vujić (FSUNS), 1m+1f, leg. Radenković (NHMB); 11m+14f, Kopaonik, Čukara, 43°12'14.96"N, 20°50'12.95"E, 1400m, 23.v.1993, leg. Vujić (FSUNS); 1m+5f, Kopaonik, Jasle-Čukara, 43°16'36.91"N, 20°46'37.09"E, 1400-1500m, 20.v.1986, leg. Radnović, Vujić (FSUNS); 12m+4f, Kopaonik, Jasle-Čukara, 43°16'36.91"N, 20°46'37.09"E, 1400-1500m, 23.v.1993, leg. Vujić (FSUNS); 1m+1f, Kopaonik, Velika reka, 43°15'39.23"N, 20°50'5.64"E, 1300m, 23.v.1986, leg. Radnović, Vujić (FSUNS).

##### Additional material.

**Serbia**: 1f, Kopaonik, Jasle-Čukara, 43°16'36.91"N, 20°46'37.09"E, 8.vi.1998, leg. Vujić (FSUNS); 1f, Kopaonik, Jasle-Čukara, 43°16'36.91"N, 20°46'37.09"E, 20.vi.1996 (FSUNS); 3m+1f, Kopaonik, 43°15’N 20°49'59.98"E, 7.vi.1998 (FSUNS); 3f, Kopaonik, Jasle-Čukara, 43°16'36.91"N, 20°46'37.09"E, 16.vi.2012, leg. Vujić (FSUNS); 1m+1f, Stara planina, Dojkinci 2, 43°15'0.07"N, 22°46'36.07"E, 01.v.2017, leg. Vujić (FSUNS); **Montenegro**: 1f, Orjen, Vratlo, 42°30'33.06"N, 18°33'25.53"E, 1.vi.2011, leg. Vujić (FSUNS); 1m, Durmitor, kanjon Sušice, 43°9'29.68"N, 18°59'39.22"E, 21.vi.1998 (FSUNS); 8f, Durmitor, Aluge, 43°8'51"N, 19°15'20.00"E, 5.vi.2016, leg. Vujić, Veličković (FSUNS); 1m, Durmitor, Krecmani, 43°8'26.08"N, 18°59'53.00"E, 22.vi.1998 (FSUNS); 1m, Durmitor, jezerska površ, 43°8'46.00"N, 19°5'33.00"E, 21.v.1998 (FSUNS); 1m, Durmitor, kanjon Komarnice, 43°0'13.00"N, 18°57'3.99"E, 21.v.1998 (FSUNS); 1m, Durmitor, Luke, 42°42'55.02"N, 19°70"E, 1.vi.1994 (FSUNS).

##### Distribution.

High mountains of the Balkan Peninsula (Fig. 10).

#### Merodon neolydicus

Taxon classificationAnimaliaDipteraSyrphidae

Vujić nom. n.

Figs 1 , 2B , 3B , 4D , 5A , 5C , 6A , 7A , 8D , 10 , 19A

##### Note.

New name for *M. lydicus* Hurkmans in an unpublished manuscript, cited in Milankov et al. (2008b) and Miličić et al. (2018); *M. lydicus* is here designated a nom. nud.

##### Diagnosis.

Dark species with broad abdomen. Oral margin only slightly notched (Fig. 2B). Basoflagellomere small, 1–1.2 times as long as broad (Figs 4D, 8D). Legs usually dark, except for pale knees. Metafemur with less developed apical triangular processes, only the apical thorn is distinct (Fig. 5A, C). Terga mostly black, terga 2 and 3 each can have small reddish lateral fasciae or maculae; transverse pair of distinct narrow microtrichose fasciate maculae on terga 2–4, approx. 1/8 of tergal length, which in some specimens can be absent from all terga (Figs 6A, 7A). Males: eye contiguity 9-12 facets long (Fig. 3B). Male genitalia with smooth thecal ridge, posterior surstyle lobe with parallel margins (Figure 1). Similar to *M. cabanerensis*, from which it differs by its larger size (10-13 mm for *M. neolydicus* nom. n. and 8-11 mm for *M. cabanerensis*), the shape of the male genitalia (Figs 1 and 9), and its distribution (*M. cabanerensis* is found in the Western Mediterranean and *M. neolydicus* nom. n. occurs in the Eastern Mediterranean).

##### Examined material.

**Type locality: Greece**: Chios, Kato Fana, 38°12'27.72"N, 25°55'37.2"E.

##### Type material.

Holotype: male, pinned, in WML. Original label: Chios, Kato Fana, 38°12'30.01"N, 25°55'43.81"E, 3.iv.2010, leg. M. J. Taylor.

Paratypes: **Israel**: 2 specimens, Jerusalem, Bet Hakerem, 31°46'31.72"N, 35°10'0.44"E, 17.iii.1951, leg. O. Theodor (NMNL); 1 specimen, Jerusalem, Wadi Ruaz, Bet Hakerem, 31°46'31.72"N, 35°10'0.44"E, 24.iii.1951, leg. O. Theodor (NMNL); 1 specimen, Jerusalem, Wadi Ruaz, Bet Hakerem, 31°46'31.72"N, 35°10'0.44"E, 10.iii.1957, leg. O. Theodor (NMNL); 2 specimens, Jerusalem, 21.iii.1929, leg. Y. Tapukin, det. *Lampetia hirsuta* by Sack (BMNH); 1 specimen, Jerusalem, Mountain Scopus, 15.vii.1930 (NMNL); 1m, Monfort, 33°2'35.15"N, 35°11'5.40"E, 10.iii.1981, leg, Frieidberg (TAU); 1f, Haifa, Har Carmel, 32°45'59.60"N, 35°1'19.027"E, 23.ii.1995, leg. J. A. W. Lucas (NMNL); 1m, Haifa, Har Carmel, 32°45'59.60"N, 35°1'19.027"E, 27.ii.1995, leg. J. A. W. Lucas (NMNL); 1f, Haifa, 32°45'59.60"N, 35°1'19.027"E, 26.ii.1977, leg. A. Freidberg, det. *Merodon hirsutus* by Hurkmans (TAU); **Greece**: 1m, Chios, Armolia, 38°16'20.28"N, 26°2'40.2"E, 3.iv.2012, leg. M. Taylor (AEU); 1f, Chios, Ag. Pateres, 38°22'19.92"N, 26°3'2.52"E, 24.iv.2002, leg. M. J. Taylor (M. Taylor coll.); 1f, Chios, Kato Fana, 38°12'27.72"N, 25°55'37.2"E, 30.iii.1998, leg. M. J. Taylor, det. *Merodon species 2* by C. Palmer (C. Palmer coll.); 6m, Chios, Kato Fana, 38°12'30.01"N, 25°55'43.81"E, 28.iii.2010, leg. M. J. Taylor (WML); 2m, Chios, Kato Fana, 38°12'30.01"N, 25°55'43.81"E, 26.iii.2010, leg. M. J. Taylor (WML); **Iran**: 1m, Yasug-Kakan, 30°35'58.99"N, 51°49'3"E, 2100m, 1-2.v.1996, leg. M. Hradsky (FSUNS); 1m, Esfahan, Semirom, Dolat Gharin, 31°32'5"N, 51°36'20"E, 2710m, 11.v.2007, leg. E. Gilasian (E. Gilasian coll.); 1m, Dascht Arajan, 38°30'29.99"N, 46°35'11"E, 20.iii.1965, leg. Saf. (FSUNS); **Syria**: 1m+1f (other information missing) (NHMW); **Turkey**: 1m, Antalya, Finike, 36°23'52.11"N, 30°6'57.87"E, 50-100m, 7.iv.1964, leg. Guichard, Harvey (BMNH); 1m, Demirkazik, Kayseri, 37°49'0"N, 35°10'0"E, 12-13.vi.1993, leg. M. Hull (C. Palmer coll.); Amanus, 1914, leg. Tölg (NHMW); **Lebanon**: 1m, Hammana, Lebanon Mountains, 33°49'45.03"N, 35°42'54.81"E, 15.v.1953, leg. G. A. Mavromoustakis (KBIN); 1m, Hammana, Lebanon Mountains, 33°49'45.03"N, 35°42'54.81"E, 16.v.1953, leg. G. A. Mavromoustakis (KBIN); 1f, Beyrouth, 19.v.1953, leg. G. A. Mavromoustakis (KBIN).

##### Additional material.

**Turkey**: 1f, Afyon, Suhut, 38°31'52"N, 30°32'45"E, 1000m, 9.v.1995 (FSUNS); 1m, Mardin Province, Mardin, 37°18'2"N, 40°45'32"E, 22.iv.2000 (NMNL); 1m, Nigde, 37°49'59.99"N, 34°45'0"E, 16.v.1961, det. *Merodon tricinctus* by Hurkmans (NMNL); **Israel**: 1f, Zova, 31°46'35.49"N, 35°7'7.205"E, 31.iii.1974, leg. M. Kaplan, det. *Merodon hirsutus* by Hurkmans (TAU); 1m, Beit Guvrim, 31°36'11.09"N, 34°54'3.94"E, 29.iii.1992, leg. A. Freidberg (TAU); 1m, Jerusalem, Wadi Ruaz Bet Hakerem, 31°46'31.72"N, 35°10'0.44"E, 29.iii.1952, leg. O. Theodor, det. *Lampetia hirsuta* by P. H. van Doesburg (NMNL); 1m, Lahav, 31°21'51.90"N, 34°51'17.96"E, 27.ii.1974, leg. A. Freidberg (TAU); 1m, Meged coastal plain, 32°1'46.87"N, 34°57'52.58"E, 27.i.1956, leg. O. Theodor, det. *L. annulata* by Doesburg (TAU); 2m, Meged, 32°1'46.87"N, 34°57'52.58"E, 27.i.1951, leg. O. Theodor (TAU); 1m+1f, Meged coastal plain, 32°1'46.87"N, 34°57'52.58"E, 27.i.1951, leg. O. Theodor, det. *L. annulata* by Doesburg (TAU); 1f, Lahav, 31°21'51.90"N, 34°51'17.96"E, 26.iii.1978, leg. A. Freidberg, det. *Merodon alexei* by Hurkmans (TAU); 1m, HaDarom, Beersheba, 31°6'0"N, 34°38'60"E, 24.iii.1954, leg. O. Theodor, det. *Lampetia hirsuta* by P. H. Doesburg (NMNL); 1m+1f, Haifa, Mount Carmel, 32°43'43"N, 35°2'48"E, 24.ii.1995 (NMNL); 21m+2f, Haifa, Mount Carmel, 32°43'43"N, 35°2'48"E, 22-27.ii.1995 (NMNL); 1m, Mount Hermon, 32°43'34.10"N, 35°18'57.14"E, 1450m, 22.iv.1973, leg. D. Furth (TAU); 1m, Hazafon, Nahal Bezet, 33°4'59.9"N, 35°6'0"E, 22.iii.1974 (NMNL); 1m, N. Bezet, 33°4'59.9"N, 35°6'0"E, 22.iii.1974, leg. D. Furth (TAU); 1f, Jerusalem, Wadi Ruaz, Bet Hakerem, 31°46'31.72"N, 35°10'0.44"E, 22.iii.1952, leg. O Theodor, det. *Merodon natans* by Hurkmans (TAU); 1m, Jerusalem, Wadi Ruaz, Bet Hakerem, 31°46'31.72"N, 35°10'0.44"E, 22.iii.1952, leg. O. Theodor (TAU); 3m, Jerusalem, Wadi Ruaz, Bet Hakerem, 31°46'31.72"N, 35°10'0.44"E, 22.iii.1952, leg. O. Theodor, det. *Lampetia hirsuta* by P. H. van Doesburg (NMNL); 2m, Jerusalem, Bet Hakerem, 31°46'31.72"N, 35°10'0.44"E, 21.ii.1953, leg. O. Theodor, det. *Merodon natans* by Hurkmans (TAU); 3m, Jerusalem, Wadi Ruaz, Bet Hakerem, 31°46'31.72"N, 35°10'0.44"E, 21.ii.1953, leg. O. Theodore, 2 m det. *Lampetia hirsuta* by P. H. Doesburg (NMNL); 1m, Jerusalem, Meged, 32°1'46.87"N, 34°57'52.58"E, 21.i.1951, leg. O. Theodor (TAU); 1m, Mount Hermon, 32°42'34.10"N, 35°18'57.14"E, 1700m, 17.v.2009, leg. A. Freidberg (TAU); 5m, Hai Tanu, 15.iii.1975, leg. M. Kaplan, A. Freidberg (TAU); 4m, Tanuri, 15.iii.1975, leg. F. Kaplan (TAU); 1m, W. Faria, 15.ii.1979, leg. D. Furth (TAU); 1m, Hazafon, Montfort, 33°2'60"N, 35°13'60"E, 14.iii.1985 (NMNL); 1m, Montfort, 33°2'60"N, 35°13'60"E, 14.iii.1985, leg. A. Freidberg (TAU); 1m, Kefar Menahem, 31°43'46.45"N, 34°50'57.54"E, 11.iii.1993, leg. A. Freidberg (TAU); 1f, Hazafon, Montfort, 33°2'35.15"N, 35°11'5.40"E, 10.iii.1981 (NMNL); 8m+2f, Montfort, 33°2'35.15"N, 35°11'5.40"E, 10.iii.1981, leg. A. Freidberg, F. Kaplan (TAU); 1f, Carmel, 32°43'43"N, 35°2'48"E, 10.iii.1979, leg. R. King, det. *Merodon aeneus* by Hurkmans (TAU); 1m, Jerusalem, Wadi Ruaz, Bet Hakerem, 31°46'31.72"N, 35°10'0.44"E, 10.iii.195? (last digit of the year is unreadable in the specimen record) leg. O. Theodor, det. *Merodon natans* by Hurkmans (TAU); 1m, 8.iii.1975, det. *Merodon hirsutus* by Hurkmans (TAU); 1m, Montfort, 33°2'35.15"N, 35°11'5.40"E, 6.iii.2000, leg. A. Freidberg (TAU); 1m+1f, Nahal-kziv, 33°1'59.98"N, 35°13'35.99"E, 5.iii.2008, leg. L. Friedman, A. Fridberg (TAU); 1m, W. Faria, 5.iii.1973, leg. M. Kaplan (TAU); 1m, Montfort, 33°2'35.15"N, 35°11'5.40"E, 4.iii.1993, leg. A. Freidberg (TAU); 1f, N. Oren, 31°56'31.79"N, 34°58'17.26"E, 4.iii.1975, leg. F. Kaplan, det. *Merodon natans* by Hurkmans (TAU); 2m, N. Oren, 31°56'31.79"N, 34°58'17.26"E, 4.iii.1975, leg. F. Kaplan, M. Kaplan (TAU); 1f, Jerusalem, Bet Hakerem, Curum 10 km Bouchir, 28°58'30"N, 50°50'17"E, 3.iii.1951 (NMNL); 2m, Bet Hakerem, 31°46'31.72"N, 35°10'0.44"E, 3.iii.1951, leg. O. Theodor, 1m det. *Merodon natans* by Hurkmans (TAU); 1m, Wadi Ruaz, Bet Hakerem, 31°46'31.72"N, 35°10'0.44"E, 3.iii.1951, leg. O. Theodor, det. *Lampetia annulata* by P. H. Doesburg (NMNL); 4m, Jerusalem, Wadi Ruaz, Bet Hakerem, 31°46'31.72"N, 35°10'0.44"E, 1.iv.1953, leg. O. Theodor, 3m det. *Merodon natans* by Hurkmans (TAU); 1m+1f, Jerusalem, Wadi Ruaz, Bet Hakerem, 31°46'31.72"N, 35°10'0.44"E, 1.iv.1953, leg. O. Theodor, det. *Lampetia hirsuta* by P. H. Doesburg (NMNL); 1m, Jerusalem, 1.iv.1953, det. *Lampetia* by P. H. Doesburg (NMNL); **Iran**: 1f, Curum, 10 km from Bouchir, 28°58'30"N, 50°50'17"E, 24.ii.1995, det. *Merodon tricinctus* by Hurkmans (NMNL); 1m, Fars, Kakan, Yasug, 30°35'58.99"N, 51°49'3"E, 2.v.1996 (NMNL); 1m, 1.iv.1936, det. *Merodon murina* (MZH); **unknown country**: 1f, 21.v.1904, leg. Aihalad (MZH).

##### Description.

***Male.*** *Head* (Figs 2B, 3B): Antenna (Figure 4D) brown to black, basoflagellomere 1.1–1.2 times as long as wide; arista dark brown. Vertical triangle isosceles, 2.2 times longer than eye contiguity. Vertex and face covered with whitish microtrichia, except for shiny oral margin. Ocellar triangle equilateral, black pilose. Frons with pale yellow pile. Eye contiguity approx. 9–12 facets long. Eye pile mostly pale.

*Thorax*: Scutum and scutellum black with bronze lustre, covered with dense, erect gray-whitish or yellow pile. Scutum with barely visible 2 longitudinal microtrichose vittae. Wing hyaline with dark-brown veins, and densely covered with microtrichia, except basal edges of cells BM and CuP. Femora and tibiae brown-black, except for paler knees and base of tibiae; tarsi dark brown dorsally (except for usually paler tarsal segments on pro- and mesolegs), light brown ventrally. Pile on legs yellow. Metafemur (Figure 5A) slightly curved.

*Abdomen*: Terga 2 and 3 can have small reddish lateral triangular fasciae or maculae; terga 2-4 each with more or less distinct white transverse fascia of microtrichia interrupted in the middle (lacking in some specimens); pile on terga erect and whitish yellow on lateral sides, but terga 2 and 3 medially with adpressed black pile, except for white pile on microtrichose bands (Figure 6A).

*Male genitalia* (Figure 1): Anterior surstyle lobe bent inwards (Figure 1B), with ventral margin slightly convex (Figure 1A); median parts of surstylus with one inner thorn (Figure 1B); posterior surstyle lobe wide and triangular, pointed apically (Figure 1A). Hypandrium wide, with smooth thecal ridge (Figure 1C).

***Female*** (Figs 5C, 7A, 8D). Similar to the male except for typical sexual dimorphism and shiny frons with narrow line of microtrichia along eye margin, mostly covered with black pile. Postalar callus and postpronotum can be yellow-red or brown.

##### Etymology.

The epithet *lydicus* is Latin, meaning “from Lydia”, and refers to the region of origin of the holotype, viz. western Turkey, which once was included in the Kingdom of Lydia and *neo* refers to the new name for this species known from unpublished manuscript. It is to be treated as an adjective.

##### Distribution.

Species distributed in the Eastern Mediterranean and Iran (Figure 10).

#### Merodon murorum

Taxon classificationAnimaliaDipteraSyrphidae

(Fabricius, 1794)

Figs 2C , 3C , 4C , 5B , 5D , 6B , 7B , 8C , 10 , 12 , 18B

Merodon auripilus Meigen, 1830: 354 **(syn. n.)**
Merodon murorum is redescribed and a neotype for M. auripilus is designated. Moreover, M. auripilus is considered a junior synonym of M. murorum.
Syrphus murorum Fabricius, 1794:

##### Type locality of *M. murorum*.

North-West Africa [as “Barbaria”], historically a region that included Morocco, Algeria, Tunisia, and Libya.

**Type material of *M. murorum*.** Holotype: male, pinned, in ZMUC. Original label: “*S. murorum*”.

##### Type locality of *M. auripilus*.

Morocco, Essaouira [as “Mogador”].

##### Type material of *M. auripilus*.

Holotype: Type presumably lost. Neotype: designated here, identified by Sack as *M. auripilus*: **Algeria**: 1m, Foret de Baïnen, 36°47'56.06"N, 2°58'20.11"E, 17.vi.1910, leg. Dr. J. Bequaert, det. *M. auripilus* by Sack (MNHN).

##### Examined material.

**Additional material. Algeria**: 1m, Santa Cruz, Oran, 35°42'24.71"N, 0°39'46.35"W, leg. Dr. J. Bequaert, det. *M. auripilus* by Vujić 2008 (MNHN); 1f, Saint-Charles, 35°42'8.10"N, 0°40'37.27"W, 1902, leg. A. Thery, det. *M. auripilus* by Vujić 2008 (MNHN); 1m, Foret de Baïnen, 36°47'56.06"N, 2°58'20.11"E, 17.vi.1910, leg. Dr. J. Bequaert, det. *M. auripilus* by Vujić 2008 (MNHN); 1f, det. *M. algirus*=*albifrons* by Sack (ZMHB); 1m, 9.x.1893, leg. A. E. Eaton (NMNL); 1f, Maison Carreé, 36°41'0.40"N, 3°8'26.50"E, leg. Dr. J. Bequaert (MNHN); 1f, 9.x.1893, leg. A. E. Eaton, det. *M. auripilus* by Hurkmans 1990 and by Vujić 2005 (NMNL); 1f, iv.1908, leg. W. Rothschild, det. *M. auripilus* by Hurkmans (BMNH); 1f, Rivet, 36°37'9.99"N, 3°13'31.00"E, 10.v.1951, leg. K. M. Guichard, det. *M. rufus* by Hurkmans (BMNH); 1f, 12.iv.1898, leg. G. Ricardo, det. *Merodon rufus* by Hurkmans (BMNH); 1m, W. Tlemcen, Khemis, Rhar el Khal, 36°17'12.59"N, 2°13'42.39"E, 10.iv.1981 (NMNL); 1m, Constantine, Constantine, 36°22'9.87"N, 6°33'45.33"E, 9.vi.1895 (NMNL); **Tunisia**: 1m, (ZMHB); m, det. *Lampetia auripila* (MNHN); 1f, Jundubah, 25 km SE Ain Draham, 36°41'50.22"N, 8°39'47.39"E, 10-16.v.1988, det. *M. auripilus* by Vujić 2008 (ZMUC); 1m, Jundubah, 40 km from Jendouba, 36°31'14.09"N, 8°41'30.32"E, 17.v.1988 (ZMUC); 1m, Hergla, salt lake south of Hergla, 35°56'57.84"N, 10°31'38.20"E, 8.iv.1988, leg. R. Schouten, det. *Merodon auripilus* by Hurkmans (NMNL); **country unknown**: 1m, det. *M. auripilus* by Vujić 2008 (MNHN).

##### Diagnosis.

**Figure 12.**
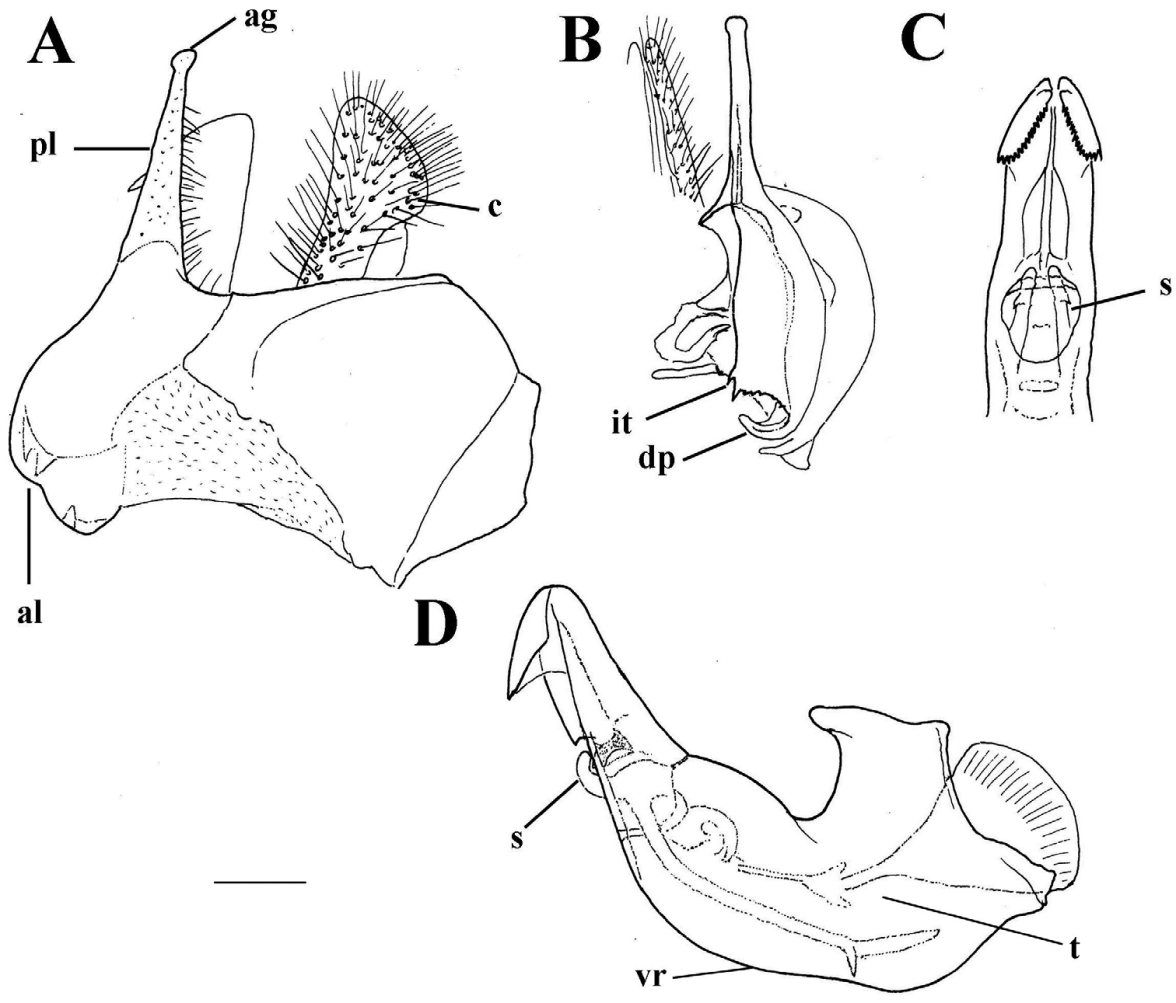
*Merodon murorum* male genitalia. **A** Epandrium, lateral view **B** Epandrium, ventral view **C** Part of hypandrium, ventral view **D** Hypandrium, lateral view, lateral view; **ag**-apical globule, **al**-anterior surstyle lobe, **pl**-posterior surstyle lobe, **c**-cercus, **s**-lateral sclerite of aedeagus, **it**-inner thorn on medial part of surstylus, **dp**-distal prolongation on anterior surstyle lobe, **vr**-ventral ridge of surstylus, **t**-theca. Scale bar: 0.2 mm.

Reddish species with long pale pile on the lateral sides of terga (Figs 6B, 7B). Terga 2 and 3 each with reddish triangular transverse maculae (Figs 6B, 7B). Basoflagellomere orange-brown (Figure 2C). Metafemur with strong apical triangular process (Figure 5B). Males: eye contiguity approx. 12 facets long (Figure 3C). Male genitalia: posterior surstyle lobe narrow and very long, with small apical globule (Figure 12). Differs from other species of the Palaearctic line by the reddish colour of the terga and the long pile along the lateral sides of terga.

##### Redescription.

***Male.*** *Head* (Figs 2C, 3C): Antenna (Figure 4C) yellow to brown, basoflagellomere 1.3–1.5 times as long as wide; arista brown. Vertical triangle isosceles, twice as long as eye contiguity. Vertex covered with dense whitish microtrichia. Face with sparse microtrichia, except for shiny oral margin. Ocellar triangle equilateral, black pilose. Frons with pale pile. Eye contiguity approx. 12 facets long. Eye pile pale.

*Thorax*: Scutum and scutellum black with bronze lustre, covered with dense, erect yellow or whitish pile. Wing hyaline with dark-brown veins, and dense microtrichia. Femora brown-black, knees and most of tibiae (or at least both ends) yellow-red; tarsi usually yellow dorsally and light brown ventrally (in some specimens all tarsi can be brown). Pile on legs yellow. Metafemur thick, slightly curved (Figure 5B) and covered with long pile.

*Abdomen*: Lateral sides red-orange to red-brown, medially black; terga 2 and 3 can have reddish triangular vittae or maculae; terga 2–4 each with more or less distinct white transverse fascia of microtrichia interrupted in the middle (lacking in some specimens) (Figure 6B); pile on terga erect, whitish yellow and very long on lateral sides; terga 2-4 medially with adpressed pile, variable in colour (from all black except for white pile on microtrichose fasciae to predominantly pale).

*Male genitalia* (Figure 12): Anterior surstyle lobe bent inwards (Figure 12B), with ventral margin slightly convex (Figure 12A); median parts of surstylus with one inner thorn (Figure 12B); posterior surstyle lobe wide and triangular, pointed apically (Figure 12A). Hypandrium wide, with smooth thecal ridge (Figure 12D).

***Female*** (Figs 5D, 7B, 8C). Similar to the male except for typical sexual dimorphism; face shiny, almost lacking microtrichia; frons shiny, with distinct line of microtrichia along eye margin. Scutum usually with two lateral and three medial longitudinal microtrichose vittae.

**Distribution.** Species distributed in North Africa (Figure 10).

Below, we redescribe an Afrotropical species of the *Merodon desuturinus* species group that is morphologically closely related to *M. desuturinus*.

#### Merodon cuthbertsoni

Taxon classificationAnimaliaDipteraSyrphidae

Curran, 1939

Figs 13A, C, D , 14

##### Type material.

Holotype: male, in AMNH. Original label: Zimbabwe, Sanyati Valley S. Rhodesia, 9–10.1925, leg. R. H. R. Stevenson, det. Curran.

##### Diagnosis.

**Figure 13.**
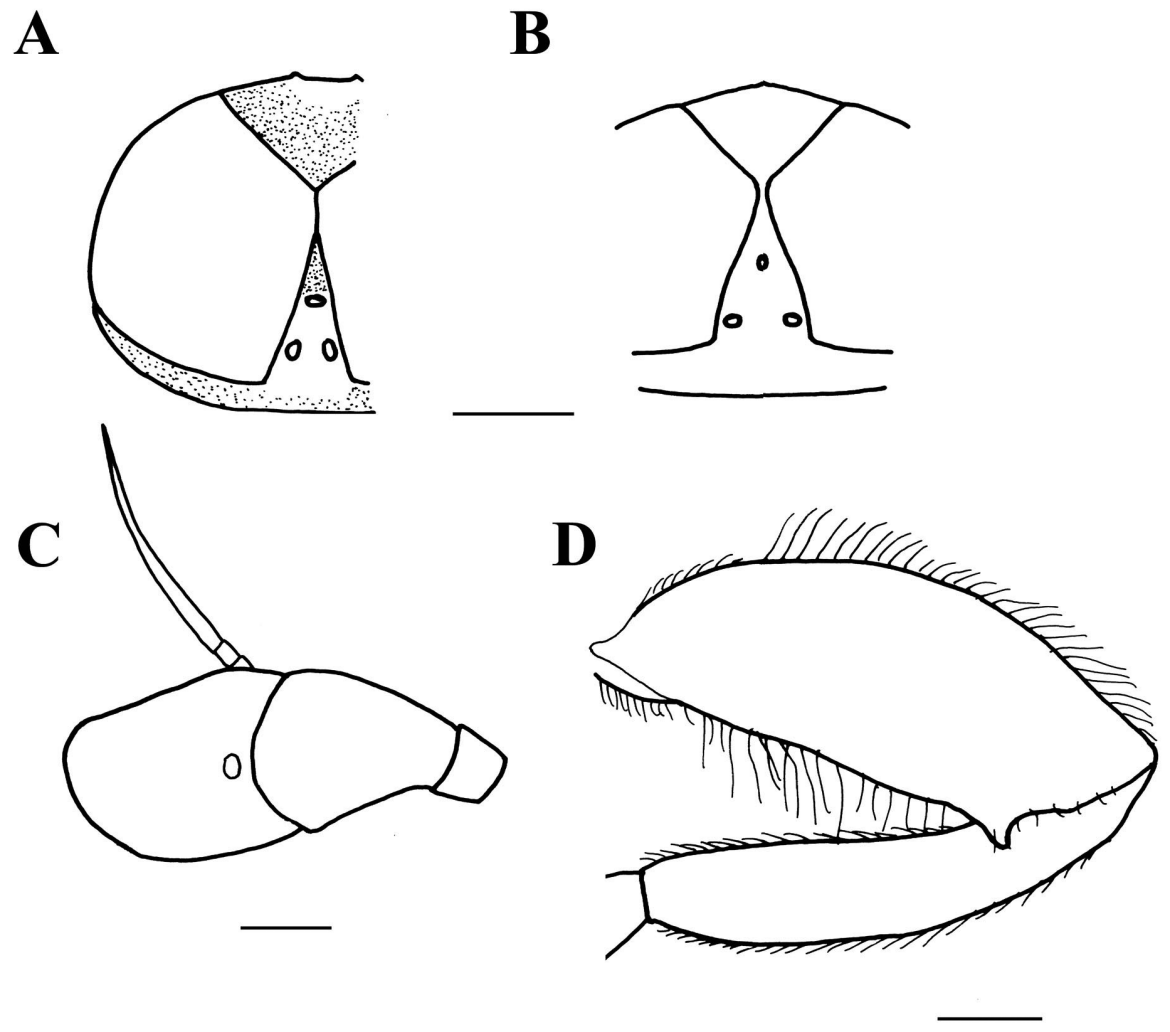
*Merodon cuthbertsoni*, holotype, male. (**A, C–D**) *Merodon desuturinus*, male (**B**): **A, B** head, dorsal view **C** antenna **D** metaleg. Scale bars: 1 mm (**A**, **B**), 0.2 mm (**C**), 1 mm (**D**).

**Figure 14.**
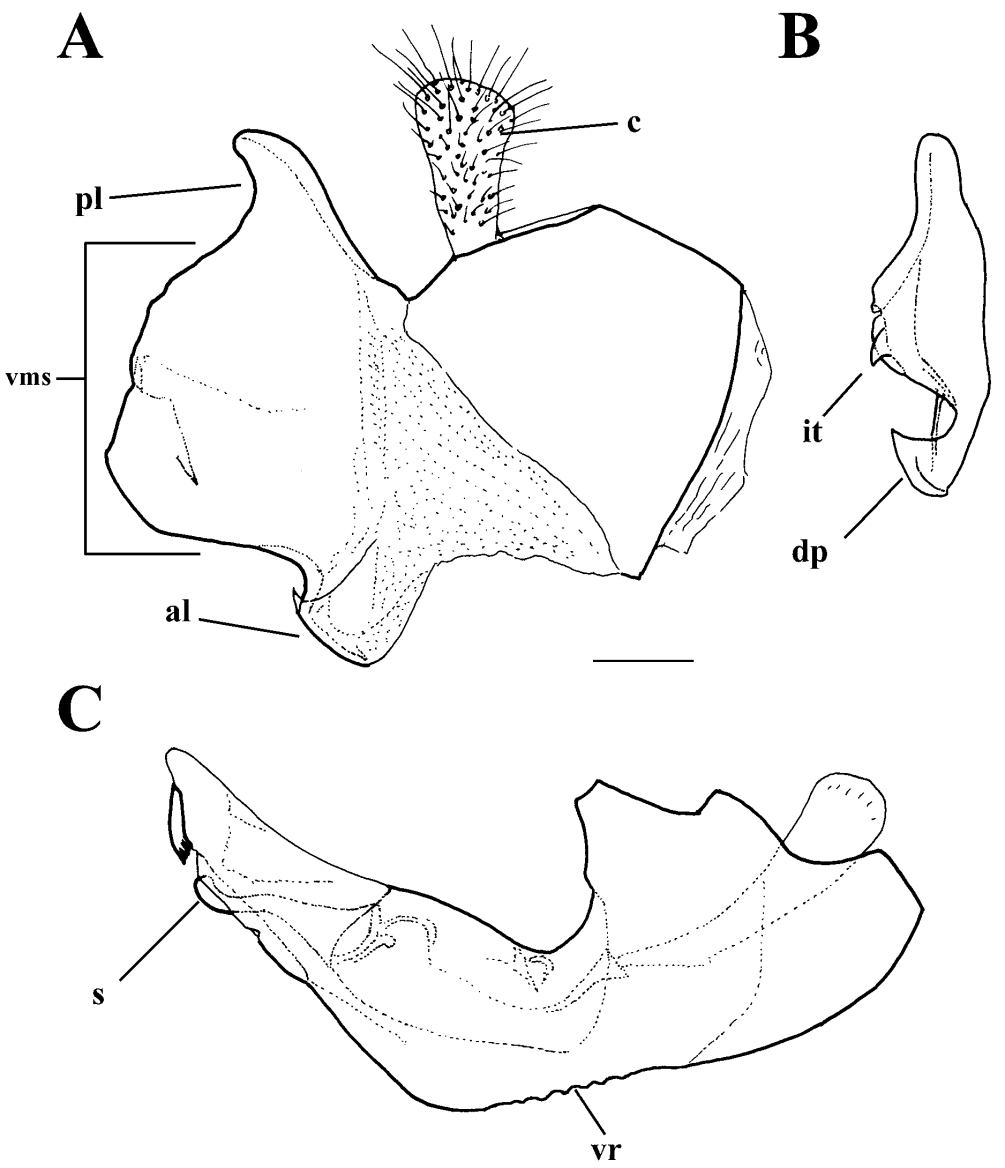
*Merodon cuthbertsoni*, holotype, male genitalia. **A** Epandrium, lateral view **B** Epandrium, ventral view **C** Hypandrium, lateral view. Abbreviations: **al**-anterior surstyle lobe, **pl**-posterior surstyle lobe, **c**-cercus, **s**-lateral sclerite of aedeagus, **it**-inner thorn on medial part of surstylus, **dp**-distal prolongation on anterior surstyle lobe, **vms**-ventral margin of surstylus, **vr**-ventral ridge of theca. Scale bar: 0.2 mm.

Face covered with microtrichia; black terga without lateral orange maculae, and terga 3 and 4 each with very narrow microtrichose fascia, approx. 1/10 of tergal length. Morphologically related to the species *M. desuturinus* from which it can be distinguished by the following features: eye contiguity is approx. 8 facets long (Figure 13A) (the eyes are separated in *M. desuturinus*, Figure 13B); tarsi entirely pale (dark brown dorsally in *M. desuturinus*); male genitalia: posterior surstyle lobe with narrow apex pointed upwards (Figure 14A) (triangular in *M. desuturinus*, Figure 11A); hypandrium with ventral margin of theca angled and folded (Figure 14C) (rounded and unfolded in *M. desuturinus*, Figure 11C). *M. cuthbertsoni* is known only from Zimbabwe (Sanyati Valley in southern Zimbabwe), whereas *M. desuturinus* is endemic to just a few high Balkan mountains (Europe).

##### Redescription.

***Male.*** *Head* (Figure 13A, C): Antenna (Figure 13C) brown, basoflagellomere 1.1 times as long as wide; arista brown and thickened basally and dark brown apically, 1.3 times longer than basoflagellomere, covered with short, dense microtrichia. Face and frons black, covered with long whitish yellow pile and sparse silver microtrichia. Oral margin shiny black, slightly protruded. Vertical triangle isosceles (Figure 13A), three times longer than eye contiguity, shiny black except in front of anterior ocellus that has white microtrichia, covered with long whitish yellow pile. Ocellar triangle slightly isosceles. Eye contiguity approx. 8 facets long. Eye pile as long as scape, pale. Occiput with whitish yellow pile, along the eye margin with dense white microtrichia and posteriorly with metallic bluish greenish lustre.

*Thorax*: Scutum and scutellum black with bronze lustre, covered with dense, erect yellow pile. Pleuron covered with grey-green microtrichia and the following parts with long yellow pile: posterior part of anterior anepisternum, posterior anepisternum (except anteroventral part), anepimeron, metasternum, and anterior, posterodorsal and posteroventral parts of katepisternum; katatergum with short, dense, erect, light-brown pile. Wing hyaline, with dense, brown microtrichia. Calypter pale yellow. Haltere with light brown pedicel and yellow capitulum. Femora dark brown-black, except for usually paler apex; tibiae dark brown with pale basal and apical parts; all tarsi yellow. Metatrochanter without processes. Metafemur (Figure 13D) thickened and slightly curved.

*Abdomen*: Black with bronze reflections, as long as mesonotum. Terga 2-4 each black with more or less distinct white transverse fascia of microtrichia, interrupted in the middle; pile on terga erect and yellow, except for central parts of terga 2-4 that are covered with adpressed black pile. Sterna blackish brown, covered with long pale yellow pile.

*Male genitalia* (Figure 14): Posterior surstyle lobe narrow, pointed upwards (Figure 14A); ventral margin of surstylus convex (Figure 14A); anterior surstyle lobe bent inwards (Figure 14B); median part of surstylus with one inner thorn (Figure 14B); cercus elongated (Figure 14A). Hypandrium with folded thecal ridge (Figure 14C: vr) and angular ventral margin (Figure 14C). Lateral sclerite of aedeagus narrow, gradually tapering, with the tip curved downwards (Figure 14C).

***Female.*** Unknown.

**Distribution**. Species endemic to Zimbabwe.

### Identification key for species of the *Merodon desuturinus* group

**Males**

**Table:** 

1	Eyes connected (Fig. 3C)	**3**
–	Eyes separated or almost touching (Figs 3A, D, 13B)	**2**
2	Basoflagellomere elongated, 1.5 times as long as wide (Fig. 4A); male genitalia: posterior surstyle lobe very short, broad and triangular (Fig. 15A) (Republic of South Africa)	***Merodon flavocerus* Hurkmans**
–	Basoflagellomere shorter, 1.1 times as long as wide (Fig. 4B); male genitalia: posterior surstyle lobe elongated and triangular (Fig. 11A) (Balkan Peninsula)	***Merodon desuturinus* Vujić, Šimić & Radenković, 1995**
3	Oral margin reduced, covered by microtrichia (Fig. 2A) (central and southern Africa)	***Merodon planifacies* subgroup**
–	Oral margin notched, slightly produced forward	**4**
4	Male genitalia: hypandrium with folded thecal ridge (Figs 16A, 14C)	**5**
–	Male genitalia: hypandrium with smooth thecal ridge (Fig. 12D)	**6**
5	Scutum with fascia of black pile between wing bases; male genitalia: ventral margin of surstylus (Figs 15B) and hypandrium convex (Republic of South Africa)	***Merodon capensis* Hurkmans**
–	Scutum entirely with pale pile; male genitalia: ventral margin of surstylus and hypandrium angular (Fig. 14C) (Zimbabwe)	***Merodon cuthbertsoni* Curran, 1939**
6	Male genitalia: posterior surstyle lobe with parallel margins (Figs 1A, 9A)	**7**
–	Male genitalia: posterior surstyle lobe triangular or with hook-like apex (Figs 12A, 15C, D, E)	**8**
7	Small species (8-11 mm) with narrow abdomen (Fig. 6D); male genitalia: ventral margin of anterior surstyle lobe angular (Fig. 9A), distal prolongation on anterior surstyle lobe broad (Fig. 9B); apical part of hypandrium narrow (Fig. 9C) (Western Mediterranean)	***Merodon cabanerensis* Marcos-García, Vujić & Mengual, 2007**
–	Large species (10-13 mm) with broad abdomen (Fig. 6A); male genitalia: ventral margin of anterior surstyle lobe rounded (Fig. 1A), distal prolongation on anterior surstyle lobe narrow and opened towards central line of symmetry (Fig. 1B), apical part of hypandrium broad (Fig. 1C) (Eastern Mediterranean)	***Merodon neolydicus* Vujić, nom. n**.
8	Male genitalia: posterior surstyle lobe long and narrow (Figs 12A, 15C)	**9**
–	Male genitalia: posterior surstyle lobe broad and triangular (Fig. 15D, E)	**10**
9	Male genitalia: posterior surstyle lobe with small apical ridge (Fig. 15C); anterior surstyle lobe evident, strongly produced forward; theca in apical fourth broad with oval lateral lamellae and small lateral wings (Fig. 16B) (Republic of South Africa)	***Merodon drakonis* Vujić & Radenković**
–	Male genitalia: posterior surstyle lobe without apical globule (Fig. 12A); anterior surstyle lobe less evident (Fig. 12A); theca without lateral lamellae or lateral wings (Fig. 12D) (North Africa)	***Merodon murorum* Fabricius, 1794**
10	Tergum 2 with orange lateral maculae; male genitalia: anterior surstyle lobe with almost straight ventral margin (Fig. 15D) and large inner thorn (Fig. 15D); theca narrow in apical quarter (Fig. 16C) (Republic of South Africa)	***Merodon melanocerus* Bezzi, 1915**
–	Tergum 2 usually black; male genitalia: anterior surstyle lobe with convex ventral margin (Fig. 15E); theca in apical quarter broad with oval lateral lamellae and small lateral wings (Fig. 16D) (Republic of South Africa)	***Merodon commutabilis* Radenković & Vujić**

**Figure 15.**
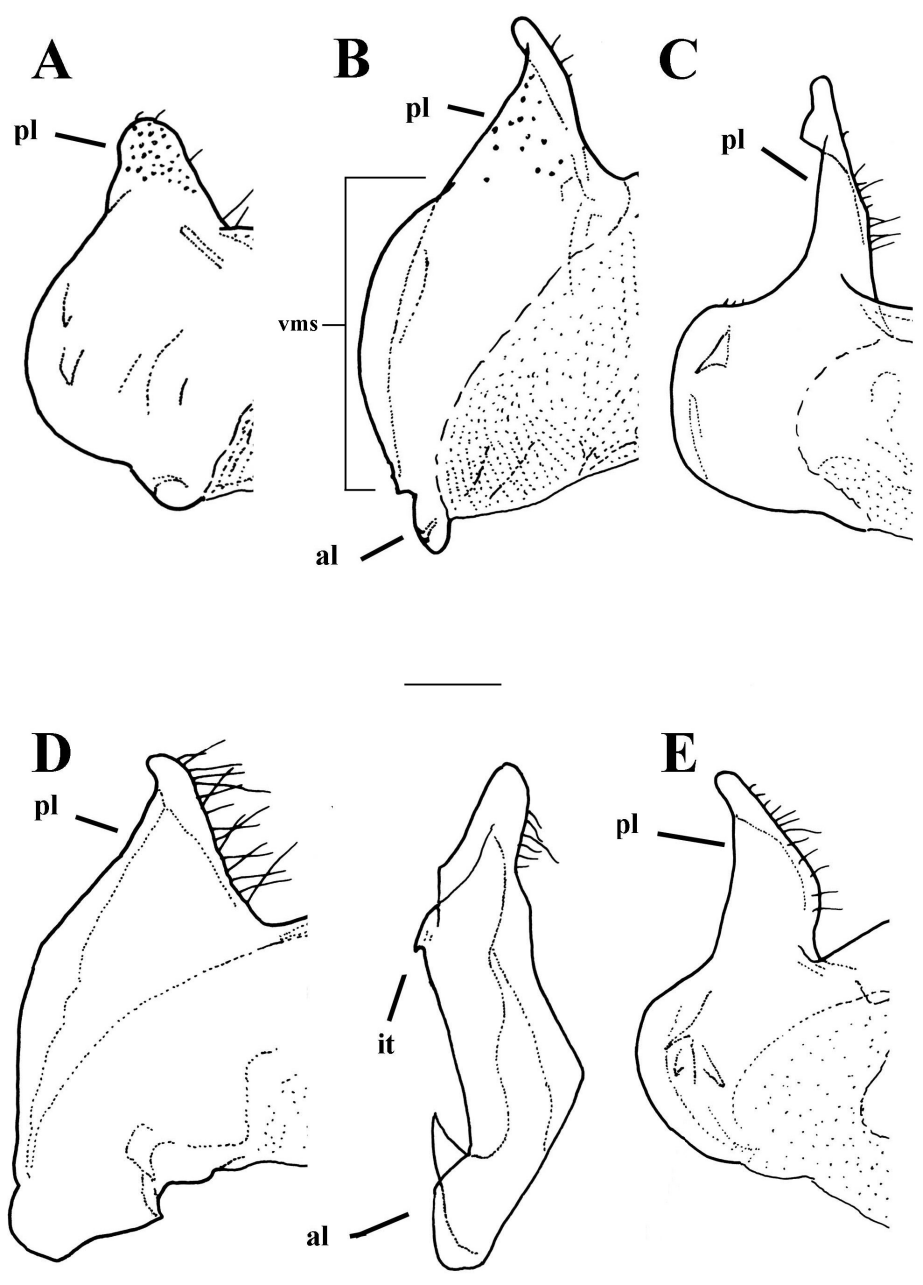
Surstylus, lateral view. **A** *Merodon flavocerus* **B** *Merodon capensis* **C** *Merodon drakonis* **D** *Merodon melanocerus* (lateral and ventral view) **E** *Merodon commutabilis*. Abbreviations: **al**- anterior surstyle lobe, **pl**-posterior surstyle lobe, **it**-inner thorn on medial part of surstylus, **vms**-ventral margin of surstylus. Scale bar: 0.2 mm.

**Figure 16.**
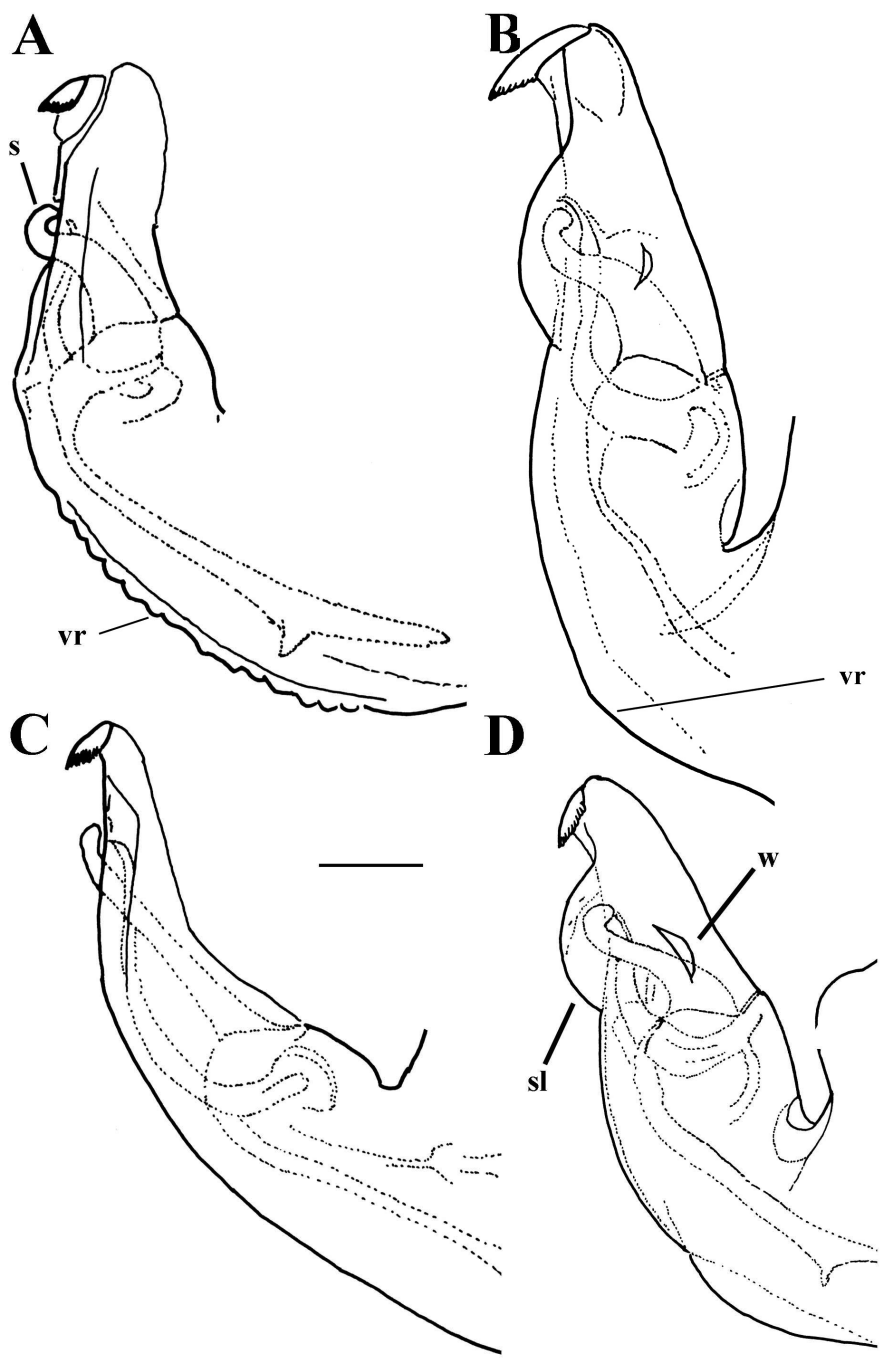
Part of hypandrium. **A** *Merodon capensis* **B** *Merodon drakonis* **C** *Merodon melanocerus* **D** *Merodon commutabilis*. Abbreviations: **w**-ventral wing of theca, **vr**-ventral ridge of theca, **sl**-subapical lamella of theca, **s**-lateral sclerite of aedeagus. Scale bar: 0.2 mm.

**Females**

(Note: female of *Merodon cuthbertsoni* is unknown, but most probably keys with *M. capensis*).

**Table:** 

1	Oral margin reduced, covered by microtrichia (Fig. 17A) (central and southern Africa)	***Merodon planifacies* subgroup**
–	Oral margin evident, notched, shiny	**2**
2	Tergum 2 black or at least lateral sides dark (Fig. 7C, D)	**3**
–	Tergum 2 with orange lateral maculae extending along lateral sides (Fig. 7A, B)	**6**
3	Legs partly pale, at least at both ends of tibiae pro- and mesolegs, and the basal tarsomeres 1–2 of pro- and mesolegs; scutum with fascia of black pile between wing bases (Republic of South Africa)	***Merodon capensis* Hurkmans**
–	Legs black, exceptionally tarsi of metalegs brown dorsally; pilosity of scutum variable, can be covered with pale or mixed black and pale pile	**4**
4	Basoflagellomere elongated, 1.3 times as long as wide (Fig. 8E); terga 2-4 each with clear microtrichose fasciate maculae (Fig. 7D) (Republic of South Africa)	***Merodon commutabilis* Radenković & Vujić**
–	Basoflagellomere shorter, 1.1 times as long as wide (Fig. 8B); terga 2-4 each with or without very narrow microtrichose fasciate maculae	**5**
5	Distribution: Balkan Peninsula	***Merodon desuturinus* Vujić, Šimić et Radenković, 1995**
–	Distribution: Western Mediterranean	***Merodon cabanerensis* Marcos-García, Vujić & Mengual, 2007**
6	Basoflagellomere elongated, more than 1.5 times as long as wide (Fig. 8A, C)	**7**
–	Basoflagellomere shorter, 1.1 times as long as wide (Fig. 8D)	**8**
7	Frons with very narrow microtrichose vittae along eye margins (Fig. 18A) (Republic of South Africa)	***Merodon flavocerus* Hurkmans**
–	Frons with broad lateral microtrichose vittae (Fig. 18B) (North Africa)	***Merodon murorum* Fabricius, 1794**
8	Body pile very short (Fig. 7A); metatrochanter with sparse pale pile (Fig. 19A) (Eastern Mediterranean)	***Merodon neolydicus* Vujić, nom. n.**
–	Body pile long (as on Fig. 19C); metatrochanter with patch of dense yellow pile (Fig. 19B); distribution: Republic of South Africa	**9**
9	Frons shiny, almost without microtrichia; distance between posterior ocelli and upper eye corner larger than distance between posterior and anterior ocellus (Fig. 17B) (Republic of South Africa)	***Merodon melanocerus* Bezzi, 1915**
–	Frons with broad lateral microtrichose vittae along eye margins; distance between posterior ocelli and upper eye corner less than distance between posterior and anterior ocellus (Fig. 17C) (Republic of South Africa)	***Merodon drakonis* Vujić & Radenković**

**Figure 17.**
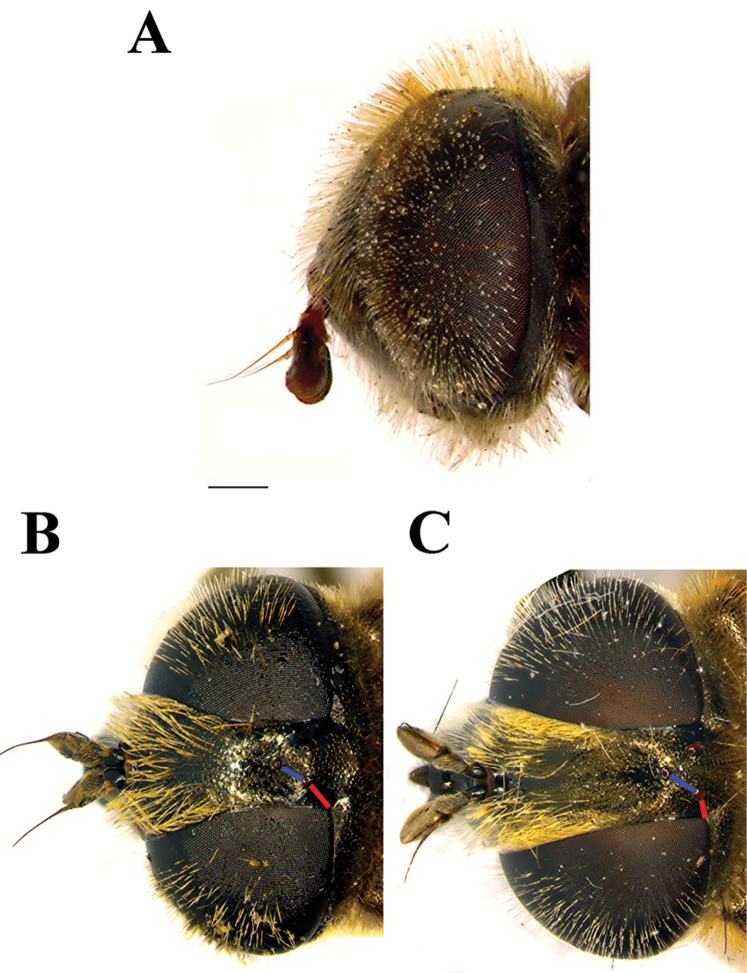
Head, female. **A** *Merodon planifacies* **B** *Merodon melanocerus* **C** *Merodon drakonis*. **A** lateral view **B–C** dorsal view. Scale bar: 1 mm.

**Figure 18.**
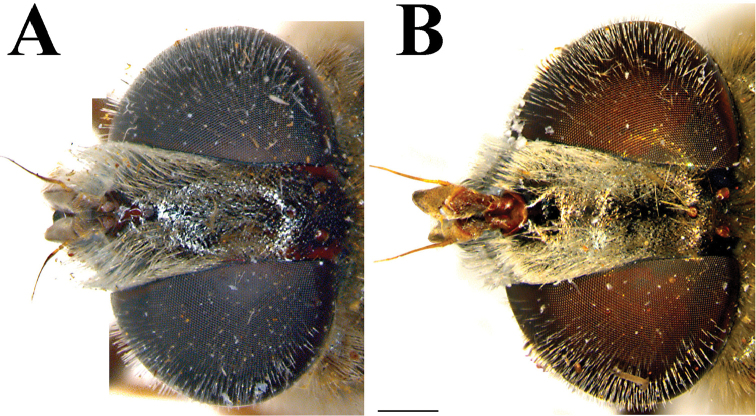
Head, female. **A** *Merodon flavocerus* **B** *Merodon murorum*. Scale bar: 1mm.

**Figura 19.**
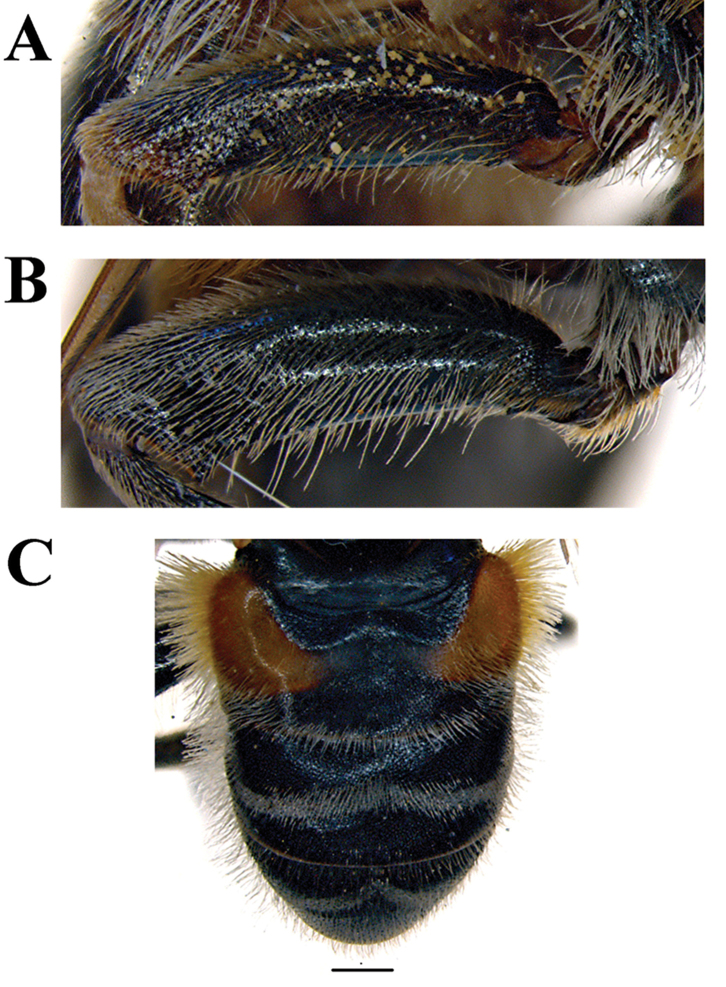
**A** *Merodon neolydicus* Vujić, nom. n. **B** *Merodon drakonis* **C** *Merodon melanocerus*. **A, B** metatrochanter, female, **C** abdomen, female. Scale bar: 1 mm.

## Discussion

The *Merodon desuturinus* clade was first mentioned by Mengual et al. (2006), and Milankov et al. (2008b) showed that this group represents an evolutionarily independent lineage among *Merodon* taxa. However, each of this study included only one species of the group (*M. cabanerensis* and *M. desuturinus*, respectively) in their analyses. Radenković et al. (2018) recently confirmed the monophyly of the *M. desuturinus* group in their analysis that examined an additional eight species of the group in relation to 27 other *Merodon* taxa, as well as its close relationship to the *albifrons* group. Based on adult morphological, molecular, and distributional data, Radenković et al. (2018) found that the *M. desuturinus* species group consists of two clearly separate lineages and represents an important link between the Palaearctic and Afrotropical faunas. They proposed that diversification in the *M. desuturinus* group most likely occurred during fundamental shifts in African climate. During the Pliocene-Pleistocene epoch, favourable conditions for *Merodon* species (increased aridity and open grasslands) in Africa most probably allowed faunal transitions from the Eastern Mediterranean (including SW Asia), with one lineage migrating to South Africa and another to the western Palaearctic.

The main morphological diagnostic character that separates these two lineages is the presence of a dense and strong yellow-to-red brush of pile on the metatrochanter in Afrotropical species, which is lacking in Palaearctic taxa. The Afrotropical lineage comprises the *M. melanocerus* subgroup of five taxa (Radenković et al. 2018), the *M. planifacies* subgroup, and the species *M. cuthbertsoni* that is morphologically related to the Palaearctic taxon *M. desuturinus*. The *M. planifacies* subgroup is characterised by a distinct apomorphic character, i.e., a reduced oral margin covered by microtrichia. *M. cuthbertsoni* is endemic to Zimbabwe, but its systematic position remains unclear. Currently, there is no genetic data on *M. cuthbertsoni* since only old museum material exists.

Our revision of the Palaearctic species from the *M. desuturinus* group has resulted in the delimitation of four species. This lineage consists of closely related yet clearly morphologically distinct species. The most distinctive species is *M. murorum*, based on the shape of the male genitalia and its reddish abdomen.

Two of these Palaearctic taxa are endemo-relicts; *M. cabanerensis* is known only from a restricted area in central Spain and Morocco and *M. desuturinus* is found only on four high mountains of the Balkan Peninsula, of which two are in Montenegro (Durmitor and Orijen) and two are in Serbia (Kopaonik and Stara planina). *Merodon neolydicus* Vujić, nom. n. is present in several countries of the Eastern Mediterranean (Greece, Turkey, Syria, Lebanon, Israel) and Iran, while *M. murorum* is distributed in North-West Africa (Algeria, Morocco, Tunisia) (Fig. 10).

Based on the distributions of the Palaearctic lineage of the *M. desuturinus* species group on high mountains of North Africa, in the Eastern Mediterranean and on the Iberian and Balkan peninsulas, they can be considered as oromediterranean relicts (Fig. 10).

## Supplementary Material

XML Treatment for Merodon cabanerensis

XML Treatment for Merodon desuturinus

XML Treatment for Merodon neolydicus

XML Treatment for Merodon murorum

XML Treatment for Merodon cuthbertsoni
